# Recent Theoretical Insights into the Oxidative Degradation of Biopolymers and Plastics by Metalloenzymes †

**DOI:** 10.3390/ijms24076368

**Published:** 2023-03-28

**Authors:** Anna Rovaletti, Luca De Gioia, Piercarlo Fantucci, Claudio Greco, Jacopo Vertemara, Giuseppe Zampella, Federica Arrigoni, Luca Bertini

**Affiliations:** 1 Department of Earth and Environmental Sciences, University of Milano-Bicocca, Piazza della Scienza 1, 20126 Milan, Italy; 2 Department of Biotechnology and Biosciences, University of Milano-Bicocca, Piazza della Scienza 2, 20126 Milan, Italy

**Keywords:** oxidative metalloenzymes, molecular modeling, biopolymers, plastic, bioremediation, biofuels, lytic polysaccharide monooxygenase, peroxidases, laccases, rubber oxygenases

## Abstract

Molecular modeling techniques have become indispensable in many fields of molecular sciences in which the details related to mechanisms and reactivity need to be studied at an atomistic level. This review article provides a collection of computational modeling works on a topic of enormous interest and urgent relevance: the properties of metalloenzymes involved in the degradation and valorization of natural biopolymers and synthetic plastics on the basis of both circular biofuel production and bioremediation strategies. In particular, we will focus on lytic polysaccharide monooxygenase, laccases, and various heme peroxidases involved in the processing of polysaccharides, lignins, rubbers, and some synthetic polymers. Special attention will be dedicated to the interaction between these enzymes and their substrate studied at different levels of theory, starting from classical molecular docking and molecular dynamics techniques up to techniques based on quantum chemistry.

## 1. Introduction

Biomass represents an abundant renewable resource that is precious for our compelling need to reduce fossil fuel depletion. Its valorization can lead to highly valuable chemicals, such as low-carbon biofuels, providing a carbon-neutral energy strategy that should mitigate the current massive accumulation of greenhouse gases [1,2,3]. For instance, plants uptake carbon dioxide (CO_2_) to grow and produce their biomass. After a plant is harvested and processed, the CO_2_ is released back into the atmosphere, where it can be absorbed by other photosynthetic organisms, closing the carbon cycle with zero net CO_2_ production. Waste biomass, in particular, is the perfect source for valorization, since it is non-edible and can be converted into what are called second-generation biofuels without interfering with food supplies [4,5]. Biomass wastes are produced daily by different sources, such as residues from forestry, agriculture, and food industry or animal, food, and municipal solid wastes [6]. Bioconversion of this non-edible biomass using microorganisms recently emerged as a promising green strategy to valorize the non-starch polymers that not only plants but crustaceans as well use to support and protect themselves [7,8].

In Nature, the most abundant raw material for biofuel production is lignocellulose, a polymeric-based biomass forming plant cell walls [9]. It is composed of polysaccharides (mainly cellulose and hemicellulose) and a highly aromatic and complex phenolic polymer, lignin, in proportions that may vary according to the type of feedstock. It has been estimated that 181.5 billion tons of lignocellulosic biomass is produced annually worldwide. Currently, 8.2 billion tons of biomass is used, of which only 1.2 billion tons is waste, suggesting that most of the high-potential of biomass waste is actually lost [10].

As for marine biomass, it contains huge amounts of the polysaccharide chitin, which is a major component of crustacean shells [11]. Global production of waste crab, shrimp, and lobster shells ranges from 6 to 8 million tonnes annually [11]. The potential utility of such seafood waste still needs to be more exploited, finding sustainable strategies to use this abundant and cheap renewable resource.

**Figure 1:**
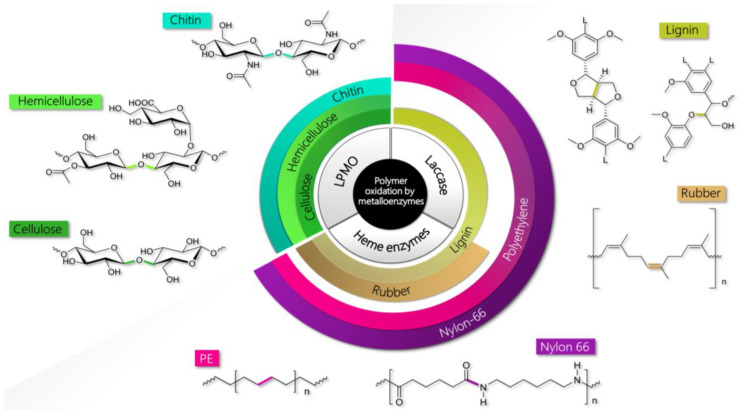
Oxidative metalloenzymes and their polymeric substrates discussed in this review.

Besides the polymeric nature, a common issue in these biomass components is their high recalcitrance towards chemical modifications. Microbes evolved to degrade such tenacious substances thanks to the action of specific enzymes, most of which are oxidative metalloenzymes, while others are hydrolytic. Within the metalloenzyme realm, the recently discovered lytic polysaccharide monooxygenases (LPMO) can oxidatively cleave sugar-based biopolymers (cellulose, hemicellulose, and chitin), while laccases and different heme peroxidases, such as lignin (LiP), manganese (MnP), and versatile (VP) peroxidases, can process lignin (Figure 1) [9,12]. The key chemical steps that are catalyzed by these enzymes all entail the oxidative breaking of the links between monomers, essentially C-C and/or C-O bonds. Interestingly, this is the same chemical action that is needed to degrade every kind of polymer, irrespective of its origin (i.e., natural vs. synthetic) [13]. It is thus not surprising that some of these metalloenzymes have been found to be capable of also degrading rubber, latex, or non-hydrolyzable plastics. Biodegradation of petroleum-based plastics and rubber by microorganisms or their enzymes provides a strategy that, together with lowering consumption and recycling, can contrast the pollution caused by the accumulation of such durable materials in the natural environment. Indeed, the rapid increase in the exploitation of hydrocarbon-based polymers during the modern age has translated into a severe disposal problem, becoming one of our most urgent environmental issues. Furthermore, biocatalysis for plastics/rubber waste degradation may allow, at the same time, recovering valuable components to be converted into other chemicals in a circular process [14,15].

In this scenario, laccases from fungi and bacteria were found to be key enzymes responsible for the biodegradation of non-polyesters plastics (i.e., resistant to hydrolysis), such as polyethylene (PE) and nylon-66 [13,16]. Similar behavior has also been ascribed to other lignin-degrading metalloenzymes, such as MnP and LiP [17]. In general, the plastic weight loss upon treatment with these enzymes can be triggered by UV pretreatment and by the presence of a redox mediator in the case of laccases [18,19]. Laccases and MnP have also been indicated as capable of degrading natural rubber [20]. This, as well as latex and vulcanized rubber, can be also oxidized and broken down by other heme peroxidases, namely rubber oxygenases (ROx) and latex-clearing proteins (Lcp) [21].

It becomes thus evident that the biotechnological potential of the above-mentioned oxidative enzymes is extremely high: besides being used to develop oxidative platforms for biomass recovery, they may also inspire environmentally friendly strategies for bioremediation [22].

For practical purposes and for productive industrial applications, optimization of these systems via protein engineering may be necessary in order to (i) increase their stability in reaction conditions that can be different from the physiological ones, (ii) tune their selectivity towards a specific catalytic task and (iii) increase as much as possible their catalytic turnovers [23]. To reach these goals in a relatively short time (for instance by rational site-specific mutagenesis [24,25]), we need a profound knowledge of structural properties (related to the metalloenzyme itself or in its interaction with the substrate(s) of interest) and of mechanistic details concerning the related catalysis. In this context, molecular modeling techniques, ranging from molecular mechanics (MM) to more sophisticated quantum mechanics (QM) or hybrid QM/MM approaches, are ideal to gain insights into the structure–activity relationships at the molecular level. Such in silico tools can thus be crucial whenever the system’s complexity may limit access to information on the active site properties, such as in the case of proteins containing transition metal-based cofactors, characterized by an intrinsically complicated chemistry.

In the present contribution, we will summarize the state of the art of computational investigations that have been carried out on metalloenzymes involved in the oxidation of both natural and synthetic polymers. The main focus will be on theoretical approaches at every level that have been used to gain information mainly regarding (i) catalytic mechanism hypothesis, (ii) substrate recognition and binding, (iii) redox properties, and (iv) electron transfer processes. We will also highlight reactivity issues that still remain essentially unexplored or poorly rationalized. The main aim is to inspire future computational or synergic theoretical/experimental attempts to optimize these biotechnologically-relevant systems towards biofuel production or bioremediation applications, or at least to collect novel knowledge regarding their functioning.

## 2. Natural and Synthetic Polymers Processed by Oxidative Metalloenzymes

Oxidative metalloenzymes and their main polymeric substrates are collected in Figure 1. In the following, we will briefly describe the essential chemical features of the polymers of interest.

### 2.1. Lignin

**Figure 2:**
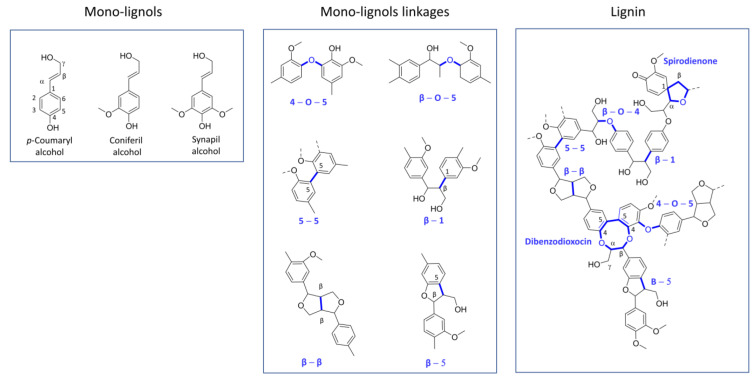
The chemical structure of the three mono-lignols. The radical–radical coupling between mono-lignols results in a variety of possible C-C and C-O ether linkages creating a complex network. The particular lignin composition in terms of mono-lignols, type of linkages, and condensed structures, such as dibenzodioxocin (5-5/β-O-4/α-O-4) and spirodienone (β-1/α-O-α), can vary from species to species and can change during the course of the plant’s growth. Baucher, Boerjan, and Ralph published comprehensive review papers in the field of lignin structure [27,30,34,35].

Lignin is an abundant natural polymer, and it is a constituent of the plant cell walls [26] accounting for 30% of the organic carbon content in the biosphere [27]. Lignin can be separated from the lignocellulosic biomass through various processes [28,29] and then moved on to its subsequent valorization. From the chemical point of view, lignin is the result of the random oxidative coupling of three 4-hydroxycinnamyl alcohols called monolignols (*p*-coumaryl alcohol, coniferyl alcohol, and sinapyl alcohol; Figure 2) through various monomer carbon–carbon and carbon–oxygen linkages (β-O-4′, β-5′, and β-β among the most common) to form an aromatic polyphenolic biopolymer that has a structural function in plants [29,30]. According to its chemical structure, lignin contains phenolic and non-phenolic substructures with the latter constituting the major part. In terms of waste biomass, lignin accounts for 15–40% of the dry biomass weight [31] and represents the largest source of renewable aromatic chemicals. Therefore, lignin biodegradation or depolymerization is the focus of intense research activity [32,33].

### 2.2. Cellulose and Hemicellulose

Cellulose dominates lignocellulose composition, being the most represented polysaccharide (and, more in general, polymer) on Earth, which plays a major structural role in lower and higher plants [36,37]. Indeed, it represents 30–50% of the lignocellulosic waste biomass [38]. Cellulose is a homopolymer of glucose, where monomers are linked by β-1,4-glycosidic bonds. Polymers, composed of 7000–15,000 glucose molecules each [38], are cross-linked by hydrogen bonds and van der Waals forces forming fibrillar architectures with crystalline properties that are highly resistant to degradation [36].

Like cellulose, hemicellulose is a plentiful polysaccharide in Nature forming 15–35% of lignocellulose waste [38]. It is a heteropolymer formed by various 5- and 6-carbon sugars, such as glucose, arabinose, galactose, xylose, and mannose, in different proportions. Unlike cellulose, it forms shorter chains (500–3000 units) that may be branched, forming amorphous structures [39]. In cell walls, hemicellulose interacts with cellulose and cross-linked lignin improving mechanical plant robustness [40]. Cellulose and hemicellulose-degrading enzymes, such as glycoside hydrolases and oxidative ones (on which this review is focused), aroused great interest within the scientific community over the past few decades since they deconstruct polysaccharides into single sugar units that are fermentable and converted into a plethora of value-added chemicals, including bioethanol [9,40].

### 2.3. Chitin

Chitin is the second most abundant biopolymer on Earth after cellulose. It is a polysaccharide that composes 15–40% of crustacean exoskeletons. It is also found in plankton and is a major component of cell walls in fungi [11,41]. Unlike (hemi) cellulose, it also contains nitrogen since it is composed of N-acetylglucosamine units. Nitrogen-containing chemicals are essential for modern life and are utilized extensively, although their industrial production requires fossil fuels and high energy inputs. Thus, chitin can provide a suitable starting point for these synthetic needs [11].

### 2.4. Natural Rubber

Natural rubber is the distinctive and primary constituent of rubber latex particles and is mostly produced by plants. It is a cross-linked polymeric substance that is extremely extensible and flexible [42]. It is composed of linear poly(1,4-isoprene) polymeric chains, each consisting of 300–10,000 C_5_H_8_ isoprene monomers that can be linked with *cis* or *trans* configuration according to the rubber-accumulating plant. Natural rubber is produced and stored in specific cells called laticifers, and its release is thought to be a defense mechanism [42]. The tire manufacturing business is responsible for over 60% of the world’s rubber use (upon vulcanization), but proper recycling of worn automobile tires into new ones (or their conversion into other industrially-relevant organic compounds) is still not possible using environmentally sustainable methods [42]. Rubber decomposition times can vary significantly from months (e.g., for rubber gloves) to 2000 years (for tires) [42].

### 2.5. Plastics

The major plastics that have been found to be decomposed by microbial action, and for whom metalloenzyme activity has been thoroughly characterized, are polyethylene (PE) and nylon-66 [16]. PE is an oil-based polymer obtained from the polymerization of ethylene monomers and, therefore, as in natural rubber, monomers are linked via C-C single bonds [42,43]. Variables including molecular weight, branching, and crystal structure affect PE mechanical properties. According to density and degree of branching, PE can be categorized as linear high-density polyethylene (HDPE), long-branched low-density polyethylene (LDPE), and short-branched linear low-density polyethylene (LLDPE). PE is one of the most manufactured and used polyolefins; its production has been predicted to reach 121.4 million tons by 2026 [44]. The primary problem of PE usage is Its extended half-life and strong environmental persistence.

As for nylon-66, it is the most widely used variety of nylon worldwide [45]. It is a thermoplastic synthetic polyamide formed by the polycondensation of two monomers: adipic acid and hexamethylenediamine (12 C atoms in total) [46]. It has durable, stiff, and elastic fibers and features very high chemical recalcitrance due to its crystalline structure.

## 3. Degradation of Polysaccharides by LPMO

### 3.1. LPMO Classification and Architecture

In 2010, a ground-breaking investigation by Vaaje-Kolstad et al. [47] highlighted a new route for oxidative cleavage of glycosidic bonds in polysaccharides by a novel class of metalloenzymes, classified at that time as CBM33 in the CAZy database and known soon after as Lytic Polysaccharide MonoOxygenases (LPMO) [48]. LPMOs can significantly boost the activity of hydrolases (such as cellulases or chitinases) and, unlike them, attack a wide range of polysaccharides not just as single polymer chains but also in their crystalline state, triggering the depolymerization process [49]. For this reason, LPMO has raised a growing interest in the context of biorefinery for both basic and applied purposes. Indeed, since LMPOs’ discovery, the number of reviews or original contributions related to their functioning and/or utilization has become impressive.

LPMOs are mono-copper enzymes that are widespread across all domains of life (among which bacteria, viruses, yeast, and fungi), and the sequences of LPMO families are continuously growing in number. Nowadays there are eight distinct “Auxiliary Activities’’ (AA) families in the CAZy database, in which LPMOs are classified according to their sequence similarity. Each AA family preferentially processes specific polysaccharides: AA9 targets both cellulose and hemicellulose; AA10, like AA15, is active on both chitin and cellulose; AA11 exclusively processes chitin; AA13 has been recently discovered to be active on starch; AA14, AA15, and AA17 target exclusively hemicellulose, cellulose, and pectin, respectively [50]. Some LPMOs can also cleave soluble polysaccharides: for instance, different AA9 LMPOs are active on cellooligosaccharides, (1→3, 1→4)-β-d-glucan(MLG), xyloglucans (XG), glucomannan, and lichenan [51,52,53].

**Figure 3:**
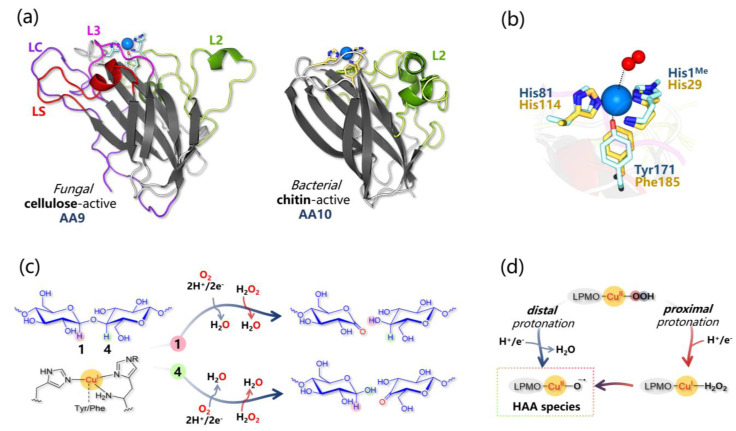
(**a**) Three-dimensional structure of a fungal AA9 (cellulose-active) and a bacterial AA10 (chitin-active) LPMO (PDB: 4EIS and 4ALC, respectively). The β-sandwich fold is in grey, binding-surface loops are highlighted in different colors, and copper is represented as a light blue sphere. (**b**) Structural detail on the active site of LPMOs depicted in panel (**a**) (AA9 in light blue and AA10 in yellow). The dioxygen molecule is only found in PDB 4EIS and water molecules are omitted. (**c**) General mechanism of LPMOs for oxidation at 1 or/and 4 positions in the presence of O_2_ or H_2_O_2_ as a co-substrate. (**d**) Calculated pathways for the formation of the Cu-oxyl species according to distal or proximal protonation of the Cu-OOH^−^ intermediate.

The architecture of all LPMOs is characterized by an immunoglobulin-like β-sandwich fold formed by nine antiparallel β-strands and varies for the content in helical and loop inserts (vide infra and Figure 3a) [50,54,55]. Interestingly, a very recent bioinformatic analysis showed that most LPMOs (except AA13) show an intrinsically disordered C-terminal region possibly serving some unknown biological function [56]. The most intriguing structural features that are almost unique to LPMOs surely are (i) the extended flat substrate-binding surface, which is found in correspondence to the Cu active site center [57,58,59,60] and (ii) the T-shaped Cu coordination, which is formed by two equatorial His ligands, one of which is an N-terminal (Figure 3b) [50,61].

This structural unit, formed by the coordination of the N-terminal His via chelation of Cu through the amino terminus NH_2_ and the π–N of the side chain, is renowned as a “histidine brace” (His-brace), and many efforts have been dedicated to relating such motif with C-H cleavage chemistry [62]. The His-brace can be N-methylated in fungal LPMOs (but not in bacterial ones) as a post-translational modification (Figure 3b). The role of His-brace methylation is not clear, and QM calculations (in the framework of the Density Functional Theory, DFT) by Kim et al. on an AA9 LPMO suggested that it should not alter the energetics of catalysis [63]. In addition, depending on the oxidation state of Cu, other ligands (e.g., Cl^−^, H_2_O, O_2_^−^) can be found in the other axial and/or equatorial coordination sites.

Other residues in the proximity of the Cu center or in its second coordination sphere are supposed to play a role in catalysis and/or in the electron hopping process: (i) the Cu ion remotely interacts with an axial Tyr in some families (such as AA9, AA11, AA13, and in some AA10, while in most AA10 LPMOs it is replaced by a Phe), which is found at quite a long distance to be considered a ligand (Figure 3b) [55]. Such Tyr has been proposed to stabilize some intermediates (vide infra) or to be part of an electron transfer chain [64,65]. (ii) A highly conserved hydrogen-bonding motif is conserved in all LPMOs’ second coordination sphere whose alteration impairs catalysis [55].

### 3.2. Brief Overview of the Catalytic Mechanism

It is generally assumed that the catalytic cycle starts upon pre-activation of the Cu(II) resting state via one-electron reduction to the catalytically competent Cu(I) ion, which shows enhanced binding affinities for substrates [66,67]. Then, catalysis requires O_2_ as a co-substrate and two electrons so that O_2_ can be activated for the scission of the glycosidic bond (Figure 3c). Electrons can be externally supplied by small molecules, such as ascorbate, gallate, and reduced glutathione, or by enzymatic partners, such as cellobiose dehydrogenase (CDH) or pyrroloquinoline-quinone-dependent pyranose dehydrogenase [68,69,70,71,72]. The nature of the co-substrate has been largely debated starting from findings reported by Bissaro and coworkers, which indicate that it can be H_2_O_2_ instead of (or together with) O_2_, shifting the LPMOs’ paradigm from oxygenases [47,52,60] to peroxygenases [73,74,75,76,77]. The main implication is that if H_2_O_2_ is the co-substrate, there is no need for electrons during catalysis, but only the first Cu(II) → Cu(I) reduction has to occur (likely prior to substrate binding) (Figure 3c).

In the past decade, since the discovery of LPMOs, intensive research has been dedicated to understanding their mechanistic details, and a significant part of this effort is represented by theoretical investigations: starting from pioneering QM (DFT) studies [63,78,79,80,81,82,83,84,85], going to QM/MM investigations [84,86,87,88,89], and finally to QM/MM/MD ones [67,89,90]. Thus, over the last ten years, several mechanistic pictures have been proposed, testing different possible intermediates for HAA from the substrate. A general consensus has been reached that a Cu(II)-oxyl (Cu(II)-O^•−^) is likely the one responsible for HAA, but different pathways for its formation have been envisioned. For example, in 2018 Bertini et al., on the basis of QM calculations based on a large-sized molecular model, indicated that Cu(II)-O^•−^ is formed upon one-electron reduction and protonation of *the distal* O atom of a Cu(II)-OOH species, followed by homolytic cleavage of the O-O bond [78]. Rovira and coworkers, instead, very recently revisited the catalytic mechanism via QM/MM/MD calculations, suggesting that O-O homolysis occurs at a Cu(I)-H_2_O_2_ intermediate formed by the arrival of one electron and one proton, which reacts with *the proximal* O of the Cu(II)-OOH intermediate [67,90] (Figure 3d). In this last case, homolysis leads to the formation of Cu(II)-OH and of a caged hydroxyl radical [86] evolving to the highly reactive Cu(II)-O^•−^ species responsible for HAA. This last mechanistic hypothesis is particularly intriguing since the formation of a Cu(I)-H_2_O_2_ species, on the one hand, naturally provides a conjunction between the O_2_ and the H_2_O_2_-dependent mechanisms; on the other hand, it rationalizes the H_2_O_2_ formation observed in the absence of substrates [73]. It is in fact reasonable that when the substrate is present the Cu(II)-O^•−^ species can be formed and catalysis occurs (coupling mechanism), while when it is absent, H_2_O_2_ is instead released into the bulk solvent (uncoupling mechanism).

Even after such a huge effort, the mechanism of LPMO remains elusive, mainly due to the lack of experimental evidence of key proposed intermediates. DFT calculations have also been used to address spectroscopic features of the active site (particularly EPR features), for example, to reproduce changes in EPR patterns upon substrate binding or upon pH variation [82,83,84,85]. Furthermore, the putative Cu-oxyl, Cu-hydroxyl, or Cu-superoxide intermediates encountered along LPMOs’ Fenton-like catalysis can exist, in principle, as low- or high-spin multiplicities [91]. This powered computational analysis at the multiconfigurational perturbation theory (CASPT2) level to address the reliability of the DFT-predicted spin-state gaps [92]. All these high-level computational works have been very recently collected in an excellent and comprehensive review by Hagemann and Hedegård, hence we address the reader to [93] for details about LPMO electronic structure, mechanistic insights, and role of the Cu second coordination sphere seen from a computational perspective.

### 3.3. LPMO-Substrate Interaction Studies

Another issue regarding LPMO reactivity that still needs to be fully rationalized is the structural basis that drives substrate selectivity and regiochemistry. Indeed, different LPMO families not only are tailored to process a sole or a few types of polysaccharides, but they also act by abstracting *specific* H atoms. In particular, some LPMOs oxidize exclusively the C1, others the C4, while others both C1/C4 in the scissile glycosidic bond (Figure 3c) [94]. Experimentally, attempts in rationalizing this different behavior among LPMOs have been made by analyzing the interactions between the enzyme and substrates by means of crystallographic, spectroscopic studies (NMR and EPR), and site-directed mutagenesis.

First, the accumulation of solved 3D structures of various LPMOs (more than 38) with known substrate specificity/regioselectivity allowed a comparison of the structural properties of their binding surfaces [54,95]. Among LPMOs, structural variability is found in the connections among β-strands that protrude in the proximity of the binding region (Figure 3a). In particular, an extended insertion composed of loops and short helix motifs is found between β-strands 1 and 3 or between β-strands 1 and 2 in AA10 and AA9 LPMOs, respectively, which is called “loop 2” or L2. The L2 sequence is highly variable among LPMOs, but it always constitutes a significant part of the binding surface, so it is supposed to influence substrate selectivity and regiochemistry. In some AA9 members, L2 contains aromatic residues (among which a Tyr residue is highly conserved) that are exposed on the surface. Another connection can be found in some AA9 enzymes between strands 3 and 4, called L3. The other two loops, called LS and LC, are found in AA9 and AA13 LPMOs, and LC in most AA9 members harbors Tyr residue(s) [57]. The involvement of AA9 surface aromatic residues in substrate binding was confirmed by mutagenesis studies [96] and the removal of a helix insert in AA9 L2, that contains the conserved Tyr can shift the C1/C4 regioselectivity to an exclusive C1 oxidation, suggesting that aromatic residues in L2 play a long range role in driving oxidation at the C4 position [65,97]. Interestingly, the presence of an insertion in the L2 of one chitin/cellulose-active AA10 LPMO (lacking in a purely chitin-active one) has been instead correlated to cellulose selectivity [65,98]. Unlike AA9 enzymes, the AA10 and AA11 enzymes are supposed to interact with the substrate mainly via polar and hydrophilic interactions [54,95] and NMR studies have also been performed to determine the residues responsible for chitin and soluble substrates binding in different LPMOs [58,59].

**Figure 4:**
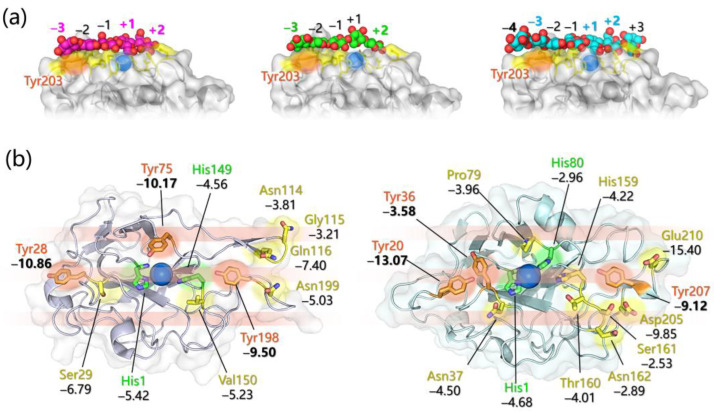
(**a**) Three-dimensional structure of three selected LPMOs in complex with oligosaccharide substrates, namely cellopentose (left, PDB 5NLS), xylopentaose (middle, PDB 5NLO), and glucomannose (right, PDB 5NKW). (**b**) Interaction energy analysis (kcal/mol) for PcAA9_D (left) and HiAA9_B (right). Calculated values have been mapped on the PDB 4B5Q and 5NNS, respectively. For HiAA9_B, only one of the two pinpointed orientations is shown, and energy values have been reported as the average over the different replicas in which the selected orientation has been obtained. Horizontal orange lines indicate the orientation of the polysaccharide chains laying on the enzyme’s flat surface.

Further insights have been gained from the crystal structure of different AA9 LPMOs in complexes with soluble substrates (Figure 4a). Cellopentaose binding to LPMO from *Lentinus similis* (LsAA9_A) revealed a key stacking interaction between the +1 sugar unit and the N-terminal His1 of the active site [60]. A similar binding pattern has been found for glucomannan oligosaccharides, and in both cases the −3 subunit stack the surface Tyr203. In contrast, the xylose at subsite +1 in xylopentaose does not stack with the His1 of the same AA9 LPMO reflecting well the observed substrate preference (Figure 4a, middle) [99]. In all these cases, the same H-bond pattern was observed between subsite +2 and residues Asn28, Asn67, and His66. Recently, similar considerations on the basis of crystallographic structures have been also carried out for *Collariella virescens* LPMO (CvAA9_A) [100].

Crystallographic and NMR studies have been combined with molecular docking simulations accounting for various oligosaccharide substrates (see Table 1 for a collection of computational studies on LPMO-substrate systems) [59,101].

The aim was to get further insights into their binding mode/energy, the possible binding sites, and the nature of residues that mainly contribute to the molecular recognition process. The simulation of the interaction between an AA9 LPMO from *Neurospora crassa* (NcAA9_C) and four ligands that were tested in biochemical essays, namely xyloglucan (XXGX), cellotetraose, cellopentaose, and cellohexaose, did not allow to conclusively determine a favorable binding orientation. In particular, the binding of the polysaccharide fragments considered resulted aspecific with no energetic preference for any site(s) over the whole protein surface, and this was ascribed to the quite low affinity for NcAA9_C towards soluble short polysaccharides with respect to substrates with higher degrees of polymerization [101]. The docking study, in this case, was inconclusive irrespective of the treatment of the ligand conformational mobility, i.e., considering them as fully flexible or fully rigid, as well as of the metal content of the LPMO model (no metal ions; one Cu^+^ center at the active site; and one Cu^+^ center at the active site plus a second Cu^+^ and a Zn^2+^ at other putative metal-binding sites in the proximity of the flat surface). This indicates that molecular docking of single-chain substrates to LPMOs may not provide insightful information unless driven by an a priori knowledge of the binding site. In fact, the inclusion of restraints derived from NMR regarding those residues more likely involved in the binding (His1, His64, Ala80, His83, and His155) in docking simulations on the same system allowed the docking of cellohexaose to NcAA9_C and to pinpoint a favorable binding site in close proximity to the active site [59]. Remarkably, the docking model was very close to the just published (at that time) crystal structure of the complex between cellohexaose and LsAA9_A featuring the +1 unit that stacks the N-terminal His (Figure 4a, left) [60]. Furthermore, L3 residues were found to be mostly involved in the binding, particularly His64, while the L2 Tyr was not intercepted by the short ligand docked suggesting that its role is crucial mainly when considering larger substrates, such as in their crystalline state.

Despite the above-listed achievements, the factors that govern both substrate selectivity and regiochemistry still need to be fully rationalized and clarified. Furthermore, many of the summarized considerations have been made on the basis of binding experimental/computational studies involving single polysaccharide chains that, however, may interact differently with LPMO if compared to the substrate embedded in a crystalline lattice (of much higher interest under a biomass-converting perspective). The substrate(crystalline)-LPMO interaction has been thus unraveled in different computational studies, since, as pointed out in [104], only computational approaches (especially the ones in the framework of molecular dynamics (MD)) allow a comprehensive visualization of the entire protein–crystalline substrate system at the atomic level. The common approach in these investigations consists in placing the crystalline substrate in proximity to the LPMO flat surface and performing MD simulations. Hence, the simulations are limited to studying the protein–substrate interactions completely neglecting the phase of protein diffusion towards the cellulose/chitin surface. The last, without the help of some enhanced sampling techniques, would be in fact not feasible due to the prohibitive simulation times required (in the order of 10^2^ μs) [102]. The most delicate step in this approach is the choice of the starting geometry since it can introduce biases in simulations by conditioning their trajectories. The common strategy was to probe different orientations of the polysaccharide chains with respect to the active site, chosen according to different rationales: (i) by analogy to previous MD investigations on a related system (i.e., the family 1 carbohydrate-binding module from *H. jecorina* Cel7A) [102,103], (ii) following indications deriving from site-directed mutagenesis and binding essays [104], or (iii) superimposing the crystalline substrate to the oligomeric one as found in enzyme–oligomer crystal structures [108]. In all the cases, the scissile glycosidic bond has been set in proximity to the Cu active center but without imposing a predetermined selectivity (C1 vs. C4) by starting from equivalent Cu-H1 and Cu-H4 distances. Another standard practice has been building an additional starting geometry by rotating the substrate by 180° in the opposite direction. In the case of cellulose, the hydrophobic 1_β_ face has always been considered as the one involved in the binding with the LPMO flat surface, while in the case of chitin, different morphologies (α- vs. β-chitin) as well as different crystallographic planes (α[100], α[110], and β[100]) have also been taken into account [104]. The main results emerging from these MD investigations are summarized in the following.

- Specific favorable orientation(s) of cellulose/chitin onto different LPMOs (both AA9 and AA10) have been detected along simulations; two examples are reported in Figure 4b. In particular: (i) in AA9 from *Phanerochaete chrysosporium* (PcAA9_D) Tyr28 (on L2) and Tyr198 (on LC) are aligned over the same cellulose chain, while the adjacent chain interacts with Tyr75 (on L3, Figure 4b, left) [102]; (ii) for AA9 from the white-rot fungus *Heterobasidion irregulare* (HiAA9_B), two different cellulose orientations have been found, a first one in which the three surface tyrosines (Tyr20, Tyr36, and Tyr207) all interact with the same cellulose chain (Figure 4b, right) and a second one in which HiAA9_B is rotated by 30° and so the Tyr residues interact with different cellulose chains [103]; (iii) in AA9 LPMO from *Myceliophtora thermophila* (MtAA9_L), the most involved residues in cellulose binding interact with two adjacent substrate chains [108]; and (iv) in chitin-active AA10 LPMO from *Serratia marcescens* (SmAA10_A), around 60–70% of contacts involves the to-be-cleaved chitin chain (considering either α- or β-chitin) [104].
- Potential energy surface (PES) for PcAA9_D-cellulose interaction revealed ~10 Å separated energy minima in correspondence to the three surface-exposed Tyr residues. This distance is compatible with the cellobiose unit, so the spacing among the aromatic residues is strategic for the polysaccharide binding, as also mentioned in other investigations [65,99,102].
- The frequency with which surface-exposed residues interact with substrates during simulations allowed determination of the important residues for binding, and interaction energy analysis allowed the quantification of the average energy contribution to the binding of each residue of interest (Figure 4b).
- During MD simulations, the cellulose/chitin slightly shifts so that the Cu-H1 distances are always shorter than the Cu-H4 ones in perfect match with the C1 regioselectivity of the LPMOs considered. This means that MD can effectively reproduce the C1 over C4 (or vice versa) oxidative preference, and thus may be used to design mutants with a desired regiochemistry.
- The SmAA10_A-chitin interaction analysis revealed the formation of a 12 Å channel between the protein and the substrate surfaces [104]. This tunnel reaches a maximum radius of ~1.6 Å suggesting that only small molecules, such as O_2_, H_2_O, or H_2_O_2_, may have access but not ascorbate or other reducing agents. It was also speculated that Glu60 may act as a gate that regulates the diffusion of substrates along the channel, which is in line with the experimental observation that Glu60 replacement causes a drop in catalytic efficiency [95].

### 3.4. Electron Transfer Investigations

The role of reducing agents along the LPMO catalytic cycle has been long discussed. Under the “classical” oxygenases paradigm, electrons should be supplied during turnover, and a first Cu(II) reduction is required independently from the co-substrate (O_2_ or H_2_O_2_). The main questions on the role of reducing agents that arose in the past decade are (i) *how do LPMO and the reducing agent interact?* (ii) *Can the reducing agent access directly the active site prior to or during catalysis?* and (iii) *if not, is there some electron transfer (ET) chain so that electrons can travel through the protein?*

**Figure 5:**
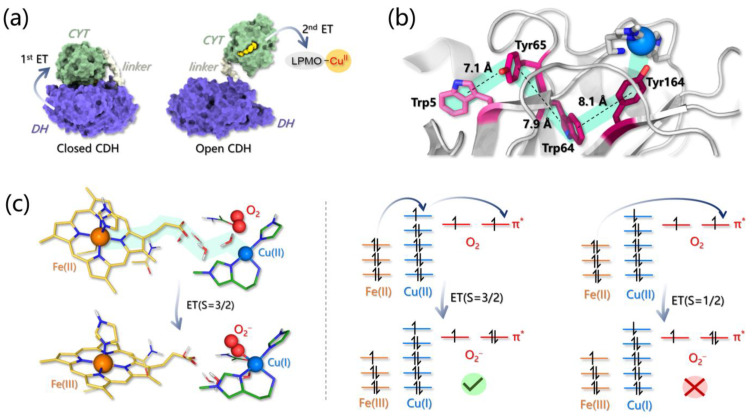
(**a**) Mechanism of the two ET from CDH to LPMO. First, the electron is transferred from DH to CYT and then from CYT to Cu(II). The structures of the closed and open conformation of CDH correspond to the PDB 4QI6 and 4QI7, respectively. (**b**) Proposed ET pathway in LsAA9 (PDB: 5ACG). (**c**) Optimized QM-portions (taken from ref [107]) of the CYT-LPMO system in the presence of oxygen before and after the ET (left) and molecular orbital occupations for the ET process in 3/2 and 1/2 state. In the 1/2 state, the electron must go directly to the O_2_ molecule and the final Fe(III)Cu(I) system is found in an open-shell singlet state, which is quite unstable if compared to the triplet one.

Several computational investigations have been carried out to answer these questions with the help of experimental evidence (and vice versa) (Table 1). As mentioned before, LPMOs can employ reducing agents of different nature, such as small organic molecules (e.g., ascorbate) or CDH. The latter is composed of a cytochrome (CYT) and a flavin-dependent cellobiose dehydrogenase (DH), and the crystal structure of the full CDH shows that these two components are connected by a long and flexible link [105]. Due to this elastic bridge, CDH can exist in two different conformations, one called “open” and the other “closed”, and the switch in conformation is fundamental for ET to occur. Starting from cellobiose oxidation at DH flavin, a first intraprotein ET occurs from the flavin cofactor to the heme group in CYT. This ET can only occur in the closed state, in which the CYT domain is docked onto the DH one, so an efficient ET is feasible. Then, upon CDH switching to the open state, CYT interacts with an external partner, such as LPMO, and transfers the electron (Figure 5a) [105].

According to a first mechanistic hypothesis, a surface-exposed Pro-Gly-Pro motif of LPMO, found at the opposite face of the active site, was proposed to be a possible docking site for CDH, entailing that ET should occur throughout the protein via tunneling and/or hole hopping in order to reach copper [65]. For instance, the redox activity of the active site Tyr in LsAA9, characterized by a combined spectroscopic–computational approach and observed under peculiar experimental conditions, was related to the participation of Tyr in different possible ET pathways linking the active site to the protein surface. The most effective one spans over ~15 Å and is formed by a second Tyr and two Trp residues (Figure 5b) [82,109].

Active site Tyr can also play a protective role by deactivating reactive intermediates formed in uncoupled turnover, as is typical for other metalloenzymes [110]. Later, a second mechanism for CDH-to-LPMO ET has been put forward on the basis of docking simulations [105,106] and NMR [105] proposing the binding of CDH to the flat surface of LPMO (i.e., in correspondence with the active site of the latter), that should promote a direct ET from the heme group of CYT to the Cu(II) of LPMO. This mechanism (in contrast with the first one) entails a binding competition between CDH and substrates that, in fact, was experimentally observed [105]. Protein–protein docking provided a first hint in favor of this second mechanism, and later, combined with MD simulations, allowed rationalizing in much more detail the basis of the CDH–LPMO interaction.

In particular, multiple docking simulations, followed by MD refinement, considering the binding of various LPMOs to different CYT types, indicated that CYT binding is much more likely to occur at the flat LPMO surface rather than far from the active site. This was supported: (i) statistically by the protein–protein docking distribution of binding modes, (ii) by the lower fluctuations, i.e., higher stability, of the LPMO-CYT complexes involving the flat LPMO surface when compared to others involving binding sites that are far from the Cu ion, and (iii) by the calculated ET rates, that are much faster if a direct heme-Cu ET can occur. It emerged that residues in close proximity to Cu in LPMOs are those mainly involved in binding and that a pivotal point in the LPMO-CYT interaction is represented by the contact between heme *b* propionate A of CYT and the Cu(II) of LPMO [105,106]. Interestingly, for almost each LPMO-CYT complex analyzed, a preferential mutual orientation of the two units has been observed. Umbrella sampling calculations were used to sample the rotation of one unit with respect to the other, revealing a broad energy minimum that reasonably reflects the transient and non-specific nature of the ET complex(es). Furthermore, this minimum seems to be mainly affected by the architecture of LPMOs, i.e., by the number of loops exposed at the binding surface, their length, and their position [106].

Computational tools have also been used to rationalize the intriguing dependence of ET from CDH to LPMO on the presence of O_2_, since the latter was found to strongly enhance ET [107,111]. First, the calculation of the ET pathway in the CHD-LPMO complex (as results from protein–protein docking experiments) indicated that it is formed by CYT heme *b* propionate A (thus confirming its critical role in LPMO reduction) and by two water molecules that were found to be essential for fast ET [107]. These residues were then included in the QM portion of QM/MM metadynamics simulations aiming at following the long-range ET from heme to Cu(II). Remarkably, if O_2_ is found in proximity to Cu(II), the ET is accompanied by the concomitant binding of O_2_ to the metal center directly forming Cu(II)-O_2_- (Figure 5c). This result indicates that the exergonic O_2_ binding can provide the driving force that is required to accelerate ET, rationalizing experimental observations. However, this holds true only if the CDH-LPMO-O_2_ system is in a quartet state since for the doublet state the ET was found to be both thermodynamically and kinetically unfavorable meaning that the process in the presence of O_2_ is intrinsically spin regulated (Figure 5c). It is interesting to notice that, according to this ET mechanism, the Cu(I) form, which is generally supposed to initiate catalysis, is completely bypassed.

For what concerns small reducing agents, such as ascorbate or gallate, bearing redox-active hydroxyl groups, they were recently supposed to actively participate in catalysis at various stages. Indeed, for instance, a full catalytic mechanism in the presence of ascorbic acid has been proposed on the basis of QM/MM/MD calculations [67]. According to this computational investigation, ascorbate (AscH^-^) first reduces the Cu(II) center to Cu(I), in a process leading to the concomitant formation of AscH^•^. The ET is predicted to be spontaneous only without invoking coordination of AscH^-^ to copper, a counterintuitive result that can be explained by the key role of water molecules found between the electron donor and acceptor in stabilizing charge separation. Subsequently, Cu(II)-O_2_^−^ is formed upon O_2_ binding to Cu(I) and it is predicted to abstract one H atom from AscH^-^ forming Cu(II)-OOH^-^ in a very facile process (ΔG^‡^ = 4.7 kcal/mol), again without AscH^-^ coordination to copper (unlike other proposed mechanisms for AscH^-^ mediated Cu-based Fenton-like chemistry) [112]. Then, according to the lowest free energy pathway (ΔG^‡^ = 7.7 kcal/mol): (i) Cu(II)-OOH^-^ performs HAA from another AscH^-^ molecule by the proximal O atom to form Cu(I)-H_2_O_2_ that, in turn, (ii) undergoes homolysis (in the absence of substrate), and (iii) the generated Cu(II)-O^•^ is prompted for HAA from the substrate.

## 4. Laccases and Lignin Oxidation

### 4.1. Classification, Architecture, and Substrates

Laccases (benzenediol oxygen oxidoreductase, EC 1.10.3.2) are blue multi-copper oxidases that catalyze the oxidation of a wide variety of substrates concomitantly with the reduction of molecular oxygen to water [113,114,115,116]. Laccases are widely distributed in bacteria [117], fungi [118], plants, and insects [119]. In Nature, their biological roles change as a function of their source of origin. Bacterial laccases are involved in spore-coating and pigmentation; fungal laccases have an important role in the lignin degradation process in wood decay; whereas plant laccases are a component of the lignin synthesizing system [120]. Moreover, laccases are considered promising enzymes for many biotechnological industrial applications [121,122] and in bioremediation [123]. Among the numerous substrates, small phenolic molecules are the ones of choice for oxidation by laccase, followed by amines, polycyclic aromatic hydrocarbons (PAHs), carbohydrates, metal compounds, and some polymers [120,124]. Due to substrate promiscuity, laccases are important in the synthesis of biologically active natural products [125,126].

Laccases are glycoproteins with monomeric, dimeric, or tetrameric structures and have a molecular mass that ranges from 50 to 140 kDa. Most of the fungal and plant laccases are extracellular enzymes with a glycosylation level ranging from 10 to 45% of the molecular mass. On the other side, bacterial laccases are intracellular with a low glycosylation level [127,128] Cupredoxin-like domains are the structural units of any multi-copper oxidase. Fungal laccases are usually three-domain enzymes of about 500 aa while bacterial laccases are two-domains (also called small laccases) of about 200 aa [117,129,130].

**Figure 6:**
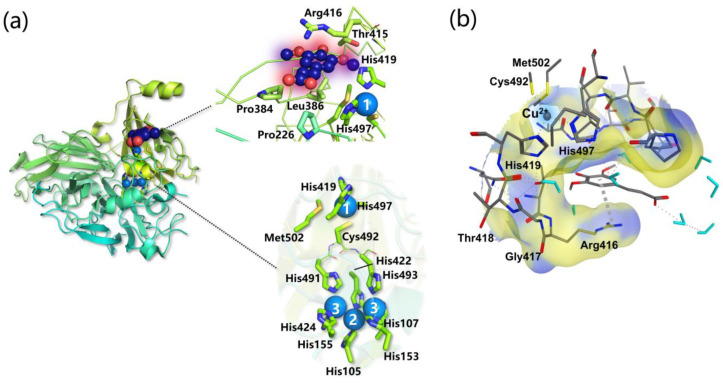
(**a**) The crystal structure of CotA laccase found in *Bacillus subtilis* complexed with sinapic acid (PDB: 4Q8B). The T1 Cu site is characterized by Met502 in apical position with SMet-Cu distance of 3.26 Å, by Cys492 with S_Cys_-Cu equal 2.20 Å and by His419 and His497 with N_His_-Cu of 2.03 Å and 2.05 Å, respectively. T1 and T2 are connected by the His491-Cys492-His493 bridge and T1-T2 distance is 12.75 Å on average. T2-T3 Cu-Cu distances are 4.13 Å and 3.69 Å. (**b**) The binding site surface around the sinapic acid.

The catalytic site is made of four copper atoms distributed at three different centers: the T1 Cu center, where the mono-electronic oxidation of the substrate takes place, and the mono-nuclear T2 and binuclear T3 centers, where O_2_ reduction occurs (Figure 6a). The T1 Cu is coordinated by two histidines, one cysteine, and an axial ligand with a distorted tetrahedral geometry [131,132]. These enzymes are also referred to as “blue laccases’’ because the intense absorption at 600 nm due to an S(π)→Cu(d_x2−y2_) ligand-to-metal charge transfer state [115] gives the blue color to the protein.

The second substrate of laccase is molecular oxygen, which reaches its binding at the T2/T3 site through a hydrophobic-type channel characterized by X-ray crystallography [133,134] and molecular dynamics [135]. This channel is also involved in the laccase inhibition by halide ions and other small moieties, as excellently reviewed by Blanford et al. [136].

**Figure 7:**
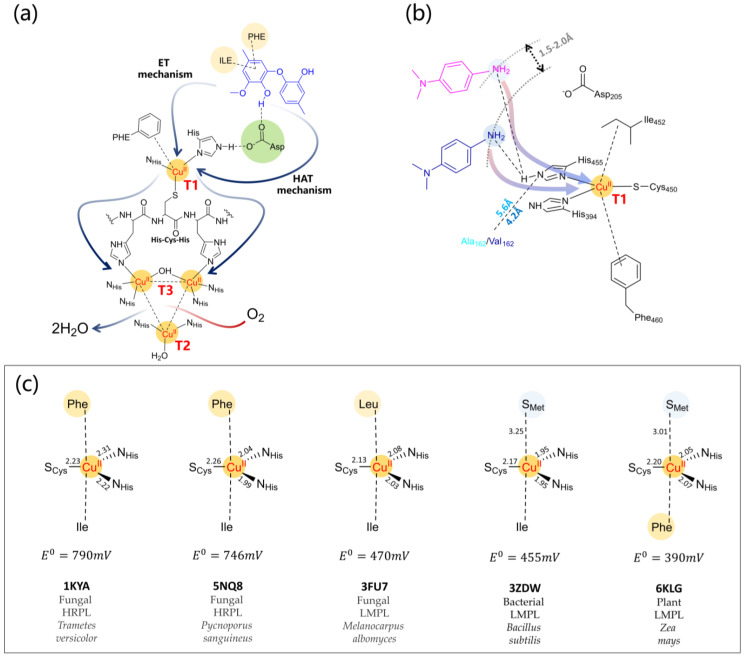
(**a**) Active site of a fungal laccase bound to the di-lignol model guaiacyl 4-O-5 dimer (in blue) as proposed in [168] considering 1KYA [147] *Trametes versicolor* laccase. The ligand is placed around 7.6 Å from the T1 Cu. Around the ligand are Phe and Ile hydrophobic interaction, the H-bond between Asp227 with one phenolic OH group. Blue arrows depict the intramolecular electron transfer from the substrate to T1 Cu and successively from T1 Cu to T2/T3 centers throughout the very conserved HCH motif. T1-T2 distance is 12–13 Å and T2-T3 3.5–4.0 Å. The substrate oxidation mechanism depends on the nature of the ligand portion under T1 attack. For phenolic portions, a H atom transfer (HAT) through Asp/Glu side chain assisted by the His residues of the T1 Cu coordination has been proposed [157,158]. For non-phenolic portions, a direct electron transfer (ET) to T1 Cu was proposed. The O_2_ channel is oriented toward one of the T3 Cu centers [121]. (**b**) Scheme of T1 Cu site of PM1 laccase and 7D5 mutant as described in [169] with the N,N-dimethyl-p-phenylenediamine ligand (in pink). The substrate forms H-bond interactions with the His455 side chain that belongs to the T1 Cu coordination and with Asp205 side chain. The V162A mutation is evidenced and belongs to the loop that defines the binding pocket. Enlarging it, the substrate approach to T1 Cu is favored (see the blue sketch of the substrate), and thus the catalytic efficiency of the mutated enzyme is increased. The distance between the nearest carbon atom of the Val/Asp side chain is taken from PDB 5ANH (PM1) and 6H5Y (7D5) [170]. (**c**) T1 coordination as a function of redox potential, spanning from fungal HRPL to fungal, bacterial, and plant LMPL. This last is referred to as the laccase from *Rhus vernificera*. Bond distances (in Å) are obtained from the corresponding PDB.

Although laccases from different organisms have nearly identical catalytic sites, they exhibit differences in redox potential, substrate specificity, and catalytic rates. The nature of the T1 Cu axial ligand is crucial to determine the potential of the enzyme. According to their redox potential (Figure 7c) at the T1 site (ranging from +400 mV in bacterial laccases with Met axial ligand to +790 mV in fungal laccases with Phe/Leu axial ligand), laccases are classified as low–medium (LMPL) and high redox potential laccases (HRPL) [137,138,139]. Interestingly, mutation of Met to Phe or Leu results in an increase of the redox potential, as observed in CotA bacterial laccase [140].

The substrate-binding cavity contains a T1 Cu site, and it is close to the enzyme’s surface. According to the analysis of the crystal structures of selected laccases, this cavity in bacterial laccases is larger than those in fungal or plant ones. Moreover, fungal laccases are HRPL with high catalytic activity, but LMPL bacterial laccases are drawing great interest due to their higher thermal/chemical stability and reactivity over a wide range of pH values [123]. Regarding this aspect, natural laccases are often not optimal for biotechnological applications due to redox potential, temperature, pH and solvent stability, and substrate specificity, although in general bacterial laccases have properties, such as stability at high temperatures and high pH, that fungal laccases do not have. However, to improve these laccase properties as required, various techniques of enzyme engineering have been considered [138,141,142,143]. Laccases are promiscuous enzymes, and this is the result of the flexible non-covalent substrate binding at the T1 Cu site. K_m_ values can vary by even three orders of magnitude as a function of their biological origin and of the substrate [144], and the molecular determinants of the substrate binding are not fully understood. X-ray crystallography on laccase-ligand structures (see Table 2 and Figure 6a,b), experimental assays [145], and computational modeling [146] evidence the role of hydrophobic side chain residues and of the exposed Asp/Glu carboxylic group.

The nature of the structural determinants that modulate the T1 redox potential has been instigated intensively but remains a challenging issue. Piontek et al. [154] observed that a longer Cu-N^His^ distance is the major factor in determining the potential of HRPL laccase: the elongation of the Cu-N bonds depletes the T1 Cu of electron density destabilizing its higher oxidation with the consequent increase of the T1 potential. Alternatively, the same effect could be achieved by increasing the T1 Cu hydrophobic environment [146]. Further investigations evidenced many second and outer coordination sphere effects that regulate the redox potential [155], such as intra-protein hydrophobic interactions, 𝜋-stacking, and H-bonding in which the metal is not directly involved.

### 4.2. Mechanistic Details of Catalysis and Redox Potential Issues Related to Substrates

The catalytic mechanism is sketched in Figure 7a and has been elucidated in detail at multiple spectroscopic and computational levels by Solomon and co-workers [115,156,157,158]. The reaction catalyzed by laccase is a four-electron process. Starting from the fully oxidized resting state, the slow step is the substrate binding at T1 Cu and its mono-electronic oxidation. For phenolic substrates, the latter step has a concerted electron/proton transfer mechanism, which avoids high-energy charged intermediates that would inevitably form if the electron or the proton were transferred individually [157,158]. The intramolecular electron transfer from T1 Cu to the T2/T3 is fast and mediated by the highly conserved His-Cys-His protein backbone. At this point, the fully reduced state binds O_2_ with the formation of the peroxy intermediate, which then evolves toward the O_2_^2−^ O-O bond cleavage with the release of water molecules. The nature of the various intermediates has been investigated also at a computational level reaching an excellent agreement with experimental data of kinetics and spectroscopy [159,160,161].

**Figure 8:**
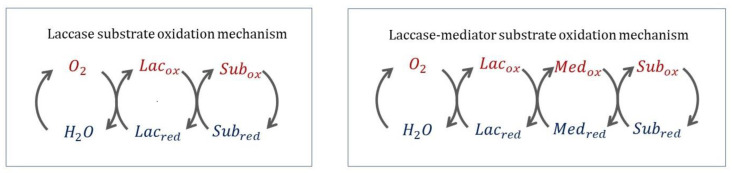
Laccase-mediated catalytic cycles for substrate oxidation. On the left: the direct substrate oxidation. On the right: the same substrate oxidation in the presence of a mediator.

Typically, substrates that are (i) small enough to approach and bind to the active site and (ii) have sufficiently low redox potential can be directly oxidized by laccase [162]. In contrast, substrates with high redox potential (such as non-phenolic and aliphatic compounds) and/or bulky substrates are recalcitrant to oxidation by laccase alone, so a *laccase mediator system* (LMS) can be employed (Figure 8). A mediator is a small molecule with a redox potential higher than that of laccase itself and with a high affinity for laccase. The mediator is oxidized during catalysis, and in turn it oxidizes the substrate. In this way, substrates with high redox potential or large size can be indirectly oxidized by laccase [163,164]. Among the substrates that cannot be oxidized directly by laccases without an LMS, we find lignin. Indeed, in the frame of lignin depolymerization, the first laccase mediator, 2,2′-azinobis(3-ethylbenzthiazoline-6-sulfonate) (ABTS), was identified [165]. Ligninolytic laccases are HRPL commonly found in white-rot and brown-rot fungi [166], and they are able to depolymerize lignin with high catalytic rates [167]. In this process, laccases catalyze one electron abstraction from the phenolic (reduction potential from 0.5 to 1.0 V vs. NHE) and non-phenolic (reduction potential higher than 1.0 V vs. NHE) portions of lignin.

In the case of the higher redox potential non-phenolic portions, LMS is necessary, which implies laccases first oxidize the mediator to form a high redox potential radical species that subsequently diffuses into the lignin realizing its depolymerization [144,171]. In the case of ABTS, the mono- and di-cationic forms are the actual oxidizing species responsible for the oxidation of the non-phenolic lignin portions [172,173,174] and even mono-lignols, such as coniferyl alcohol, can act as laccase mediators [175].

Starting from the seminal paper by Bourbonnais and Paice [165], a significant number of excellent review articles have recently been published on laccase-lignin degradation and bioconversion. The general consensus regarding this issue can be summarized as follows. Laccase-Lignin degradation in Nature is attributed to fungal laccase, mainly from white-rot fungi [166] since they are characterized by a high redox potential [176]. Bacterial laccase can also catalyze lignin degradation but to a lesser extent due to their lower redox potential, while plant laccase assist lignin biosynthesis.

As the non-phenolic portion of lignin has a potential higher than that of the enzyme, the LMS must be employed [177,178]. The mechanism of substrate oxidation (Figure 7a) has been investigated considering mono- and oligomer-lignin models both considering laccase alone and various laccase-mediator systems [179]. Due to their redox potentials, laccase can oxidize the phenolic components of lignin allowing single-electron transfer by the T1 Cu. As for the non-phenolic portion, depending on the chemical nature of the system, two different mechanisms of the mediator oxidation were observed, an electron transfer mechanism or a radical hydrogen atom transfer [180,181].

### 4.3. Computational Modeling Techniques as Valuable Tools in the Laccase-Lignin Degradation Insights

In the understanding of the laccase-lignin depolymerization, an important point is the interaction and the reactivity of the enzyme with the substrate in terms of (i) the redox potential of the substrate and enzyme; (ii) substrate steric hindrance; (iii) the nature of the mediator [177]; and (iv) the possibility of enzyme engineering. The investigation of these aspects can be supported by molecular modeling techniques at various levels, in particular by (i) classical molecular docking and dynamic to elucidate substrate–enzyme binding (Table 3); and (ii) quantum chemistry DFT or QM/MM modeling to investigate the effect of the first and second coordination sphere on T1 Cu redox potential. In general, substrate binding can be studied profitably at the classical level, whereas the oxygen reduction mechanism has received more attention at the quantum chemistry level.

After a thorough survey of the scientific literature of the last decade on the laccase-lignin modeling, several significant results emerge that can be grouped and illustrated in the following.

Various methods have been tested to verify whether the computational values of observables are in line with those determined experimentally. As an example, the crystallographic pose of a given ligand or relevant kinetic parameters have been considered. In particular:

- The reliability of the molecular docking procedure was tested to be able to properly reproduce experimentally available laccase substrate binding with ABTS, 2,6-Dimethoxyphenol, or 2,5-xylidine [192].
- It has been ascertained that observed *K*_M_ values relate directly to the lifetime of the active substrate of the enzyme and estimated binding free energies [194,195] and kinetic data correlate with the DFT spin population of the substrate, in particular, the *k*_cat_ value [32,169,215]. Reorganization energies for multi-copper oxidases can be computed at the DFT level [216] and those of laccases for the first mono-electronic ET from the substrate to T1 Cu relates inversely to the enzymatic activity [132] showing that this step could determine the laccase turnover.
- Finally, the literature shows that the full-QM cluster model and QM/MM level were able to reproduce redox potential variations of two mutants compared to the wild-type enzyme. Specifically, the redox potential (a) increases for *Escherichia coli* CueO mutant L502K [146]; and (b) decreases for the F463M mutant from *Trametes Versicolor* laccase [217].

The features of the enzymatic binding pocket that have proven to be crucial for the binding of lignin-based substrates are similar to those already illustrated above. Of particular interest are (i) a cluster of hydrophobic residues (Phe is common) [183]; and (ii) a few polar residues (Asp/Glu/His), which can be involved in hydrogen bonding interactions [191]. The substrate–enzyme binding depends on the laccase type considered. In the case of plant laccases, the stability substrate–enzyme complex decreases with the increase of the lignin model size, while the opposite holds true for fungal laccases [168,187]. Moreover, white- and brown-rot fungi laccases exhibited stronger binding affinity towards lignin model compounds compared to soft-rot fungal laccases [198]. In bacterial laccases, substrate affinity is less effective in terms of binding energy [189,210,218] and requires conformational changes of the methionine-rich region [219] that provide a binding site for exogenous copper ions. There is a link between T1 Cu redox potential, catalytic efficiency, and substrate binding pocket structure [220]. Takur et al. [190] showed that HRPL can degrade larger lignin models because of their larger binding pocket volume size, while LRPL, due to their smaller binding pocket, cannot accommodate bulky ligands. Interestingly, some laccases can bind large ligands, such as trimer and tetramer lignols, in a different binding pocket far from T1 Cu [205,206].

The mechanism of the laccase substrate binding has been investigated extensively for a variety of possible compounds. There is a large consensus that hydrophobic interactions are necessary for lignin–laccase interaction, while H-bonds would seem less essential [175,189,190,197,198,203]. Particularly, Cambria et al. [183] highlighted that the initial substrate recognition is due to a hydrophobic interaction with the enzyme, which is later stabilized by hydrogen bonding. This description is in line with the nature of amino acid residues commonly involved in the interactions between various compounds and laccases, which always include non-polar residues, in particular Phe, Ala, and Leu. Thanks to these residues, laccases can bind and oxidize non-phenolic substrates, such as polycyclic aromatic hydrocarbons [221] and textile organic dyes [222].

An interesting aspect that arises from simulations is the energy difference between catalytically productive and unproductive laccase–ligand poses [223], where these last are characterized by the absence of those interactions that trigger the first ET from the substrate to T1 Cu (in the case of phenolic substrates the H-bond with the His residues at T1 Cu coordination and with the Asp/Glu side chain). Enzyme–substrate binding modes for the ligands of choice (such as ABTS and phenolic substrates) result to be very stable as shown by molecular dynamic simulations [189,210], revealing that such thermodynamic stability is associated with a network of hydrogen bonding also mediated by water molecules [211]. In particular, the hydrogen bond between the Asp side chain and the phenolic OH group has been often observed [195] and, in the case of ABTS, positively charged residues in the pocket are important for high substrate turnover [186].

Once the substrate is bound to the enzyme pocket, its oxidation mechanism depends on the chemical nature of the substrate itself (Figure 7a). For phenolic substrates, the HAT mechanism has been proposed since the acidic proton of the OH group is removable by a suitable base. In the case of non-phenolic substrates, the ET mechanism was proposed, as in the case of polycyclic aromatic compounds [123,137,196,224]. Qualitatively, it has been observed that the catalytic activity of the enzyme depends on the distance between T1 Cu and the substrate, as reported in separate contributions by Camarero et al. and de Salas et al., in which the mutant 7D5 of a high-potential laccase from *Aspergillus oryza* (nine mutations compared to the wild type), identified through enzyme engineering [170,225], was produced and structurally characterized at XRD and QM/MM levels [169]. The data obtained indicate that catalytically productive ligand poses are numerically increased in the mutant and correlate nicely with the kinetic data. In such context, the T1 Cu site interacting with N,N-dimethyl-p-phenylenediamine (DMPD) ligand through an H-bond with the His residues is sketched in Figure 7b. Considering the DMPD ligand and T1 Cu as an electron-donor/acceptor couple, one can conclude that the shortening of the interatomic distance (X_donor_-Y_acceptor_) determines the charge transfer dynamics increasing the ET rate constant, which, in turn, results in an increase of the k_cat_ by 3.5- to 8-fold compared to the wild-type.

## 5. Heme Peroxidases for Lignin Oxidation

### 5.1. The General Structure of Ligninolytic Peroxidases

In addition to laccases, alternative enzymes responsible for the degradation of lignin in Nature belong to the heme peroxidases in class-II—e.g., secreted fungal peroxidases—of the peroxidase catalase superfamily [226], which comprises lignin peroxidases (LiP; EC 1.11.1.14), manganese peroxidases (MnP; EC 1.11.1.13), and versatile peroxidases (VP; EC 1.11.1.16). All fungal ligninolytic peroxidases are extracellular enzymes that share a similar topology and folding formed predominantly by alpha helices in two domains among which the heme group is tightly embedded. In all cases, the protein structure is stabilized by the presence of four disulfide bridges (five in the case of MnP) and by two structural Ca^2+^ ions [227].

**Figure 9:**
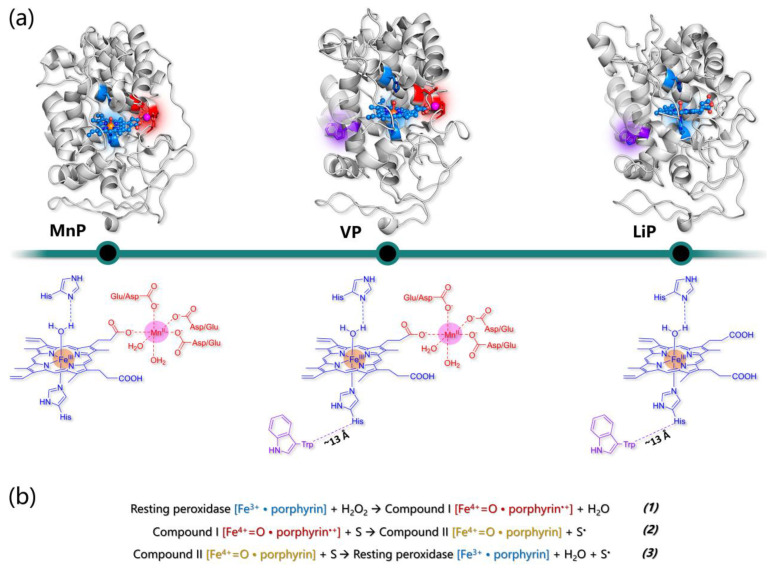
(**a**) The crystal structure of MnP found in *Phanerodontia chrysosporium* (PDB: 1YYD), VP found in *Pleurotus eryngii* (PDB: 2BOQ), and LiP found in *Phanerodontia chrysosporium* (PDB: 1B82). For each enzyme, a 2D representation of the catalytically active sites is reported. (**b**) Scheme of the catalytic cycle reactions of class-II heme peroxidases (S represents a generic substrate).

The active site of class-II heme-peroxidases is characterized by an iron protoporphyrin IX, with a high-spin Fe(III) coordinated by four nitrogen atoms of the tetrapyrrole ring by the N_ϵ_ of proximal histidine, as well as by a water molecule, which is hydrogen-bonded to a second axial histidine (distal His) (see Figure 9a). The catalytic cycle of the ligninolytic enzymes is similar to that of other peroxidases. The resting state enzyme with ferric heme is oxidized by hydrogen peroxide forming compound I (reaction 1 in Figure 9b), a two-electron oxidized intermediate containing an oxo-ferryl species (Fe(IV)=O) and a porphyrin cation radical. In this intermediate, two unpaired electrons are localized on the iron-oxo moiety, while a third unpaired electron is found on the porphyrin ring. The highly reactive enzyme intermediate compound I is then reduced by one substrate molecule to compound II (reaction 2 in Figure 9b). At this point, a further reduction back to the resting enzyme can be accomplished either by the same substrate molecule or by a second one (reaction 3 in Figure 9b).

**Figure 10:**
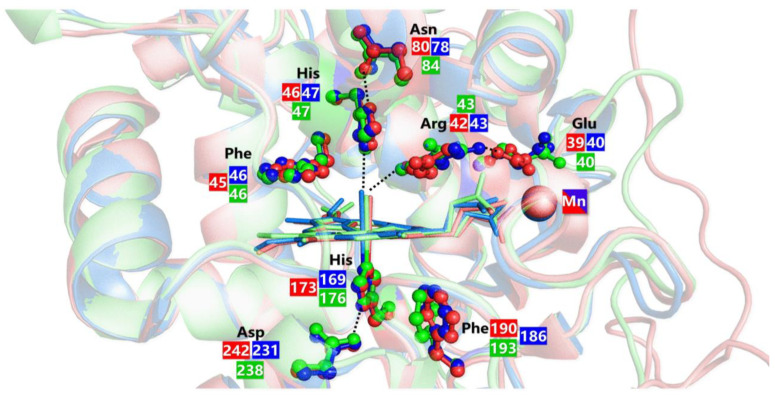
Representation of the conserved residues in the heme pocket of all ligninolytic peroxidases. MnP is represented in red (PDB: 1YYD), VP is represented in blue (PDB: 2BOQ), and LiP is represented in green (PDB: 1B82).

Apart from the two axial (proximal and distal) His, several amino acids at both sides of the heme pocket are conserved in all ligninolytic peroxidases. These include aspartate and phenylalanine at the proximal side; the former establishes an H-bond with the proximal histidine contributing to the stabilization of compound I. As for the conserved Phe, its role has not been clearly established yet. However, it was reported that its substitution decreases LiP and MnP stability. On the other side of the heme group, where coordination of hydrogen peroxide to the heme iron occurs, more conserved residues are present in all ligninolytic peroxidases evidencing their fundamental role in the peroxidative catalytic function (Figure 10).

A group of four residues composed of arginine, phenylalanine, glutamate, and asparagine surrounds the distal histidine and forms a conserved H-bond network in which the imidazole ring of distal His is deprotonated (in the resting state) thus facilitating the cleavage of the O–O bond in the peroxide–iron complex. Moreover, the Arg residue is responsible for the stabilization of the Fe(IV)=O complex thanks to the establishment of an H-bond interaction with the oxygen atom [228,229]. Other residues conserved among the ligninolytic family class are involved in the substrate oxidation, and these include three acidic residues chelating the Mn ion in MnP and VP or an exposed tryptophan related to veratryl alcohol oxidation in LiP and VP (Figure 10). The real difference among the three families of ligninolytic peroxidases—LiP, MnP, and VP—resides in the substrates that are one-electron reduced by compounds I and II.

### 5.2. Manganese Peroxidases

Mn-dependent peroxidases are unique in utilizing Mn(II) as the reducing substrate. Mn(II) is ubiquitous in all lignocelluloses and in soil, and it is bound by MnP in a specific site close to the heme active site. Only one Mn-binding pocket is present in the protein formed by a specific disulfide bond among two cysteines (Cys341–Cys348 in PDB 1YYD), which pushes the C-terminal polypeptide chain away from the main body of the protein. Interestingly, LiP—which does not allocate a Mn(II) ion—lacks the presence of this specific disulfide bridge. Mn(II) is taken into place by a heme propionate, three acidic ligands—e.g., Glu35, Glu39, and Aps179 in PDB 1YYD—and two water molecules (Figure 9a).

After the formation of compound I, it can be reduced by either Mn(II) or other electron donors, such as ferrocyanide and phenolics [230]. However, Mn(II) is required for the reduction of compound II leading to the regeneration of native enzymes and the release of the second water molecule. If the oxidizable substrate of both compound I and compound II is Mn(II), two equivalents of Mn(III) are generated for each catalytic cycle. MnP-generated Mn(III) can act as a diffusible oxidizer by mediating the oxidation of organic substrates, such as various monomeric and dimeric phenols, including phenolic lignin model compounds [231,232].

However, since Mn(III) is a strong oxidizer (1.5 V), it is unstable in aqueous solution. Therefore, organic chelators, such as malonate and oxalate, are required for allowing the dissociation of Mn(III) from the enzyme and for the stabilization of the diffusing oxidizers. The existence of such low molecular weight redox mediators is fundamental for the initial degradation of lignin since the compact molecular structure of the biopolymer prevents direct enzyme–lignin contact [227]. Therefore, Mn(III) is responsible for the direct oxidation of the minor phenolic substructures of lignin but also for the indirect oxidation of the non-phenolic lignin units through the generation of lipid peroxyl radicals.

A theoretical study using a QM model of MnP was carried out in order to shed light on the catalytic mechanism of this enzyme for the oxidation of non-phenolic compounds in the case of the broad-spectrum antibiotic sulfamethoxazole (SMX). It was reported that the reduction of compound I to compound II and to the resting state follows two proton-coupled electron transfer (PCET) reactions. Moreover, the one-electron abstraction from the SMX substrate in favor of the Mn(III) group was modeled. Since the effects of the organic acid ligands on the oxidation ability of Mn(III) was ignored, energy profiles for the multiple product reactions were only used for the qualitative analysis in conjunction with experimental results from HPLC-MS analysis. Combination of experimental and theoretical work allowed for the recognition of the most plausible products deriving from the one-electron transfer reaction [233].

The MnP family can be divided into three subfamilies identified by the length of the C-terminal tail as short, long, and extralong MnP. Differences in the protein length reflect different catalytic properties and pH stability. In fact, short MnP lacking the C-terminal tail extension (14–22-residue shorter C-tail) evidenced catalytic activity even in the absence of Mn(II). Short MnP can in fact oxidize ABTS at the Mn(II)-oxidation site. The different activity among the three MnP subfamilies was clarified by means of site-directed mutagenesis and molecular simulations, which demonstrated that the C-tail extension in long and extralong MnP surrounds the entrance of the heme-propionate channel thus limiting the access to substrates different from Mn(II) [234]. Moreover, the authors were able to report the first crystal structure of short MnP from *P. ostreatus* (PDB 4BM1) enabling future investigation and direct comparison with the alternative two MnP subfamilies [235].

Short MnPs show a higher capacity for oxidizing ABTS allowing it to reach the heme group; however, their activity at low pH (pH = 2) is immediately lost. In contrast, long MnPs retain their activity in such extreme conditions. Rational enzyme engineering of long MnP from *C. subvermispora* was carried out with the help of computational methods with the aim of obtaining an ABTS oxidizer with the pH stability properties of long MnPs. ABTS docking in wild-type and mutated MnP in association with electronic coupling calculations at the QM/MM level turned out to be successful for the development of an ABTS active and stable acidic pH peroxidase through the introduction of two specific surface mutations—two histidines plausibly involved in hydrogen bond interactions with the sulfonate groups of ABTS—placed far away from the active site [236].

Alternative computational research in MnP focused on the study of the binding interactions of this enzyme with lignin model compounds. Molecular docking studies were carried out to describe such interaction using a lignin model substrate and identified the best docking pose for the lignin model compound in the proximity of the heme pocket, with the most important residue for the binding of lignin being characterized by residues also implied in the binding of Mn(II) [236].

### 5.3. Lignin Peroxidases

Evolutionary studies show that the incorporation of a solvent-exposed tryptophan in MnP resulted in the development of the so-called versatile peroxidases. Subsequent mutations led to the loss of the Mn(II) oxidation site giving rise to all the lignin peroxidase LiP enzymes. VP constitutes the link between MnP and LiP, since it combines the catalytic properties of the above two families due to the presence of both a Mn-oxidation site and a catalytic tryptophan [237,238]. LiP represents the most efficient lignin-degrading enzyme family [239]. LiP enzymes are relatively nonspecific to their substrates and they show an exceptionally broad substrate spectrum that includes phenolic and non-phenolic lignin model compounds as well as a range of organic compounds [240,241,242,243,244] thanks to an exhibited redox potential up to 1.4 V (versus normal hydrogen electrode) in the presence of hydrogen peroxide.

The large redox potential exhibited by fungal peroxidases has been attributed to a significant increase in the distance between the iron ion in the heme group and the nitrogen atom belonging to its axial histidine ligand compared to other peroxidases, such as cytochrome c peroxidase (2.15 A vs. 1.95 A in pristine LiP and CcP from *Saccharomyces cerevisiae*). Displacement of the contiguous proximal histidine from the metal increases its imidazolate character—i.e., its basicity—thus stabilizing the high-oxidation states of the heme. Elongation of the Fe–NHis bond is a consequence of the strong H-bond interaction between the axial histidine and the contiguous aspartate residue (His176-Asp238 in pristine LiP) as well as between the serine bound to proximal histidine and another aspartate belonging to a different protein helix (His177-Asp201 in pristine LiP). This H-bond causes the movement of the proximal histidine residue away from the heme and weakens the Fe–NHis bond, thereby increasing the heme iron electron deficiency [227,238,239,245].

LiP nonspecificity towards its reducing agent is due to the fact that such an enzyme can perform electron abstraction from substrates through an oxidation site at the protein surface: electrons are then delivered to the heme cofactor of the H_2_O_2_-activated enzyme via a long-range electron transfer pathway (LRET). The oxidation site at the surface was identified to be a tryptophan residue, specifically, it is constituted by Trp171 in *P. chrysosporium* LiP (see Figure 9a). The presence of a surface catalytic tryptophan residue accelerates interaction with bulky and high-redox-potential substrates, such as lignin. Moreover, it was demonstrated that its presence is strictly required for oxidation of the non-phenolic moiety, which represents the major and more recalcitrant part of the lignin polymer [246].

**Figure 11:**
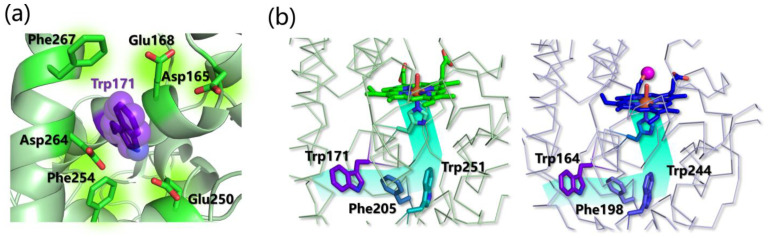
(**a**) Representation of the conserved residues surrounding the catalytically active tryptophan in LiPs (PDB: 1B82 from *P. chrysosporium*). (**b**) Residues involved in the LRET path in LiP (right, PDB: 1B82 from *P. chrysosporium*) and VP (left, PDB: 2BOQ from *P. eryngii*).

Electron transfer from Thr171 to the heme group requires the presence of a second tryptophan residue—i.e., Trp251—whose vital role in electron transfer was experimentally and theoretically demonstrated [247,248]. Acebes et al. individuated the most plausible long-rage electron transfer route thanks to the help of QM/MM calculations (see Figure 11). The Trp171–Phe205–Trp251–heme was described to be the main LRET path in LiP. Interestingly, the presence of the two tryptophan residues is constant in all the LiP sequences while phenylalanine is also highly conserved but it is, in some cases, mutated to a tyrosine residue [248].

The initial one-electron transfer from the catalytic solvent-exposed tryptophan generates a reactive tryptophanyl radical whose existence has been demonstrated thanks to electron paramagnetic resonance (EPR) studies of LiP variants (site-directed mutagenesis of the Trp171 micro-environment) coupled with QM/MM calculations [249]. Such a computational technique was needed in order to obtain a detailed description of the environment surrounding the considered neutral and cationic (protonated) Trp radicals, but most of all it was fundamental to elucidate the most stable Trp radical species by considering the nearby protein residues and solvent water molecules. It resulted that, in pristine LiP, the positive charge of the cationic species ThrH^+•^ is stabilized by the highly negative electrostatic potential generated by the protein environment surrounding the Thr171 residue.

How LiP safely manages its high oxidative power without self-destructing still represents an intriguing topic for scientists. An interesting aspect plausibly related to this regard was observed in LiP crystal structures derived from fungi, i.e., the presence of a Trp171 stereospecifically hydroxylated at its Cβ-atom [245]. Such hydroxylation is formed during the first few turnover cycles of the enzyme probably due to an autocatalytic self-oxidation reaction and it is, therefore, absent in the crystal structure of pristine LiP (LiPH8) [250]. The reaction leading to Cβ-hydroxylation is supposed to be derived from the addition of water to the indolenine structure via nucleophilic attack at the *Si*-side of the prochiral indolenine plane. The presence of the tryptophan hydroxylation in Cβ position leaves the indole ring intact and functional and it can be considered as the less invasive modification of Trp, which prevents further degrading steps that will occur in the presence of Trp radicals in solution.

Lignin-degrading heme peroxidases’ enzymatic activity exhibits pH dependency, with an observed optimal activity at a pH around 3 [239]. In physiological conditions, such acidic pH is a consequence of lignin degradation due to the secretion of organic acids by white-rot basidiomycetes; therefore, intrinsic resistance of ligninolytic enzymes under acidic conditions is a requisite. However, redox potential decrease or inactivation occur outside the narrow range of their optimal pH (pH 3–4) [251]. This characteristic is not a problem in the natural biodegradation of lignin but can represent a major limiting factor in the industrial application. Lignin degradation at low pH turned out to be fundamental because (i) it avoids repolymerization of phenolic products dimerization [252] and (ii) more acidic conditions stabilize cationic radical intermediates rather than phenoxy radical ones, thus enabling bond-cleavage reactions to occur via lower energetic paths [252].

Moreover, a recent study based on QM/MM calculations evidenced the existence of a relationship between the charge distribution around the active site with the redox potential of the compound I/compound II couple. At a low pH, even deeply buried residues can be protonated—e.g., Asp238 interacting with a proximal histidine—and this results in a destabilization of compound I with a consequent increase of the redox potential. Thanks to this study, it was evidenced that the protonation of titratable residues reflects an increase of the computed redox potential when the residue is closer to the active site, but a large effect is also observed at long range [252].

Several studies have been dedicated to the rational engineering of ligninolytic enzymes in order to design LiP with the aim of augmenting their pH stability. Among the three families of class II peroxidases, fungi-derived MnPs show the highest stability under extremely acidic conditions. For instance, extralong manganese peroxidase MnP6 from *C. subvermispora* (PDB 4CZN) maintained more than 80% of the initial activity after incubation at pH 2 for 24 h. Resistance to extreme pH conditions was rationalized as a consequence of the presence of several noncovalent interactions, such as salt bridges and a hydrogen bonding network [234]. Based on this observation, an acid-stable LiP variant was designed with the aid of in silico-based strategies through the introduction of new salt bridges in native LiPH8 (LiPH8 variant A55R/N156E-H239E and PDB 6A6Q). Interestingly, the engineered enzyme exhibited remarkable stability under extremely acidic conditions as observed for MnP6, but it still retained an exposed catalytic active tryptophan for lignin oxidation [251].

In the catalysis performed by LiP, the presence of a specific molecule turned out to be imperative for the oxidation of high-redox potential substrates [227]. Such a role is played by veratryl alcohol (VA), a secreted secondary metabolite of *P. chrysosporium* and other white-rot fungi. Moreover, it was demonstrated that VA can restore enzymatic activity when an excess of H_2_O_2_ led to the formation of inactive compound III rather than a closed catalysis cycle [253]. In the LiP catalysis, it was originally believed that substrate oxidation would occur at the heme site. However, crystallographic structures of all the lignin-oxidizing heme peroxidases exhibit a narrow heme access channel, plausibly related to the need of protecting the enzyme from inactivation by substrate radicals generated during catalysis [227]. Such a heme access channel is conserved in all heme peroxidase families and allows the entrance of hydrogen peroxide (necessary for the enzyme activation) as well as of different small substrates. Early molecular dynamic simulations of LiP protein in solution suggested that VA could approach the active site through the main access channel, and it could be accommodated at this pocket and be stabilized by specific hydrogen bonding and hydrophobic interactions [254]. Accordingly, subsequent docking and MD studies located the binding site of the lignin-model molecule ABTS at the active site of LiP [185]. Despite the presence of Trp171, which is exposed on the protein surface and was found to be necessary for VA oxidation as it was demonstrated through site-directed mutagenesis [255], the above-reported computational studies did not consider the binding of VA at the Trp171 site. Moreover, it was shown experimentally that the depletion of Tyr171 in LiP did not involve the loss of oxidative activity towards small substrates characterized by a redox potential lower than VA [255]. Such experimental observations suggest that small molecules can reach the heme active site and be directly oxidized there while VA and larger molecules—such as lignin—are most plausibly oxidized at the catalytic active tryptophan site [227,238,245]). However, the size of the molecule is not the only discriminating factor to define the possibility that it can access the heme site (see Table 4 for a collection of computational studies on heme peroxidase-substrate systems). In fact, it has recently been reported that atrazine, one of the most commonly employed herbicides in agriculture, cannot be oxidized by LiP enzymes. Protein–ligand docking and MD simulations allowed to relate the inactivity towards atrazine oxidation to the impossibility of such a molecule to enter the heme pocket because of the formation of stable interactions between the protein and the protein-ligand. These long-range interactions are established thanks to electric charges of the delocalized s-triazine ring [256].

The Trp171-surrounding environment is constituted by numerous acidic residues (Glu168, Glu250, and Asp264) as well as aromatic ones (Phe254 and Phe267) that provide a favorable site for interaction with VA (Figure 11a). Molecular dynamics simulations conducted by Recabarren et al. confirmed this assumption by showing that VA interacts at the Trp171 site by hydrogen bonding interactions with the acidic amino acids Asp264 and Glu168, as well as by hydrophobic interactions with Phe267 [257]. Similar results were obtained for LiP from *T. cervina* in which a redox-active surface exposed tyrosine was identified [258]. In this case, molecular docking showed that VA forms stable π-stacking complexes with the aromatic residue Phe89 and the active redox residue Tyr181 [259].

Further investigations addressed the binding of VA to the LiP surface through a combination of theoretical and experimental methods [260]. By means of MD, QM/MM spin density calculations, site-directed mutagenesis, and kinetic experiments, the authors were able to suggest that VA binding at the tyrosine site is energetically favored. Moreover, the authors suggest that the formation of a Trp–VA^+•^ complex is better stabilized with respect to the initial complex between VA and the Trp radical in its neutral or cationic forms (TrpH^+•^/Trp^•^).

### 5.4. Versatile Peroxidases

The third family of heme peroxidase ligninolytic enzymes is constituted by versatile peroxidase, which, as already mentioned above, combines properties of the other two families. In particular, as shown in Figure 9a, in VP a Mn(II) oxidation site and a catalytically active tryptophan residue on the protein surface (Trp164 in *P. eryngii*) coexist (Figure 9a) [261]. Nonetheless, some notable variations in the oxidation of aromatic substrates exist between VP and LiP. One example is the absence of VP* Trp164 Cβ–hydroxylation in contrast to the case of Cβ–hydroxylation involving the Trp171 moiety in both the wild-type LiP generated by *P. chrysosporium* and the recombinant enzyme from *E. coli* treated with small amounts of peroxide equivalents [245,262]. Moreover, differences in the environment of the exposed Trp residue are found between VPs and LiP. The former exhibits a less acidic environment as it is surrounded only by two (Glu161 and Glu243) of the four (Glu168, Asp165, Glu250, and Glu264) acidic residues present in LiP isoenzymes. This characteristic involves a substantial difference in the stabilization of the radical Trp, as has been demonstrated by computational analysis. In fact, contrary to what is observed for LiP [249], QM/MM results for VP indicate that the positively charged Trp-surrounding environment—due to the presence of an arginine residue Arg257 instead of an aspartic acid Asp264 in LiP—fits well in a neutral Thr^•^ radical [263].

The other significant difference between the two families relies on the higher oxidation efficiency of VA shown by LiP compared to VP. However, it was reported that VP is able to directly oxidize many other recalcitrant molecules, which are oxidized by LiP only in the presence of VA [262]. Experimental studies of VPs from *P. ostreatus* evidenced its direct lignin-degrading activity, being VP able to degrade non-phenolic lignin models by bond cleavage at the Cα–Cβ position [235]. Due to the large dimensions of lignin, the one-electron oxidation of the recalcitrant polymer must occur via a long-range electron transfer from the active surface-exposed tryptophan residue to the heme group [246]. In analogy to what was reported for LiP, the LRET in VP was mapped thanks to a combination of computational analysis with site-directed mutagenesis [248]. The LRET in VP, characterized by means of QM/MM techniques, was suggested to follow the Trp164–Phe198–Trp244–heme path (Figure 11) [248].

The catalytic promiscuity of VPs makes them appealing from an application point of view, in particular as industrial biocatalysts. However, as observed for MnPs and LiPs, a major limiting factor in the industrial application of VPs is constituted by their narrow range of in vitro activity conditions. Therefore, many studies have been dedicated to the engineering of this enzyme in order to increase the spectrum of activity at a wider range of pH and temperature [264,265,266].

**Table 4:** Summary of the literature about molecular docking, MD, QM, or hybrid studies on MnP and LiP interaction with ligninolytic substrates or ABTS.

**MnP**	**PDB**	**Docking**	**MD, QM, Hybrid**	**Ref.**
*Phanerodontia chrysosporium*	3M5Q	ABTS	MD	[185]
*Gelatoporia subvermispora*	4CZN (extralong)	ABTS	MD, QM/MM	[234]
*Phanerodontia chrysosporium*	4BM1, 4CZN	ABTS	QM/MM	[236]
*Phanerochaete chrysosporium*	1MNP	ABTS		[209]
**LiP**	**PDB**	**Docking**	**MD, QM, Hybrid**	**Ref.**
*Phanerochaete chrysosporium*	1QPA (HTR171) wt and W171A	VA	MD	[254]
*Phanerochaete chrysosporium*	1LLP	ABTS	MD	[185]
*Phanerochaete chrysosporium*	1LLP	VA	MD, QM/MM	[257]
*Phanerochaete chrysosporium*	1B82	Atrazine	MD	[256]
*Phanerochaete chrysosporium*	1LLP wt and E250Q	VA	MD, QM/MM	[260]
*Bacterial LiP*	Homology model	12 lignin models *		[267]
*Phanerochaete chrysosporium*	1LGA	ABTS	MD	[209]

* Two chlorinated lignin compounds, eight standard lignin monomers, one dimer, and one trimer.

## 6. Degradation of Rubber and Plastics by Oxidative Metalloenzymes

### 6.1. Nature Strategy for Rubber Degradation: Heme Oxygenases

**Figure 12:**
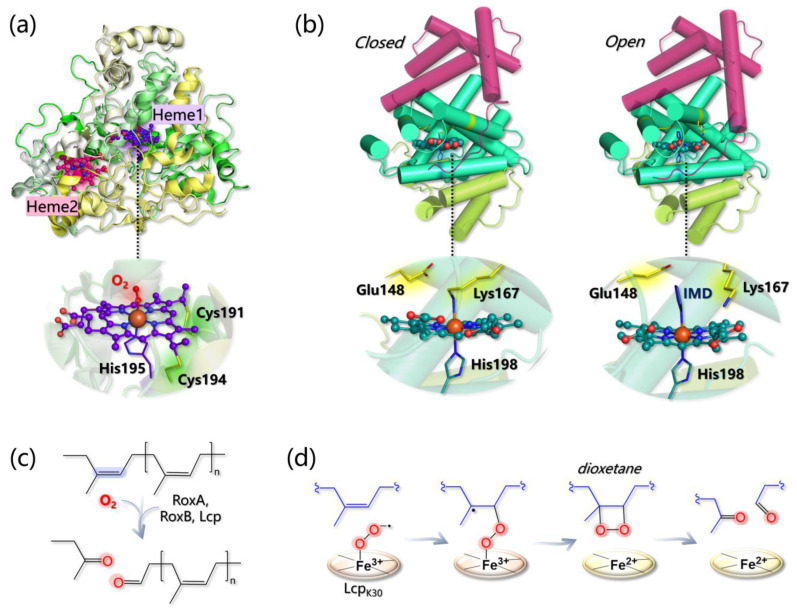
(**a**) Structure of RoxA (PDB 4B2N) and detail on heme1 coordination. (**b**) Closed (left) and open (right) conformations of Lcp (PDB 5O1L and 5O1M, respectively). IMD refers to the axial imidazole ligand of heme, that is found in the 3D structure of the open state. In both states, the globin core, containing the heme active site, is colored in green-cyan, while the other three- and six-helices domains are colored in light green and purple, respectively. (**c**) General mechanism of dioxygenation by RoxA, RoxB, and Lcp. (**d**) Energetically favored pathway as calculated in [283].

In Nature, several strains of NR-degrading bacteria exist whose activity solely relies on oxidative metalloenzymes [21,268,269,270,271]. Indeed, the lack of hydrolyzable groups makes polyisoprene completely inert toward hydrolytic enzymes. To date, three families of extracellular heme-enzymes, isolated from NR-degrading bacteria, with dioxygenase activity towards poly(1,4-isoprene) have been characterized, namely rubber oxygenases A (RoxA), rubber oxygenases B (RoxB), and latex clearing proteins (Lcp) (Figure 12a,b) [13,268,272]. RoxA and RoxB, active towards poly(cis-1,4-isoprene) (the most abundant polyisoprene form in nature), are distantly related from an evolutionary standpoint and are both found in Gram-negative rubber-degrading bacteria. In particular, RoxB can be considered a separate subgroup of RoxA homologs. Lcp, active towards both poly(cis-1,4-isoprene) and poly(trans-1,4-isoprene), is found, instead, in Gram-positive bacteria and does not share any sequence/structural relationship with either RoxA or RoxB [273].

The catalytic activity of RoxA, RoxB, and Lcp consists of the oxidation and breaking of the polyisoprene double bonds, forming oligoisoprenoids with terminal keto or aldehyde groups (Figure 12c). In general, these enzymes follow two distinct oxidation strategies that lead to different product distributions: RoxA catalyzes isoprene cleavage producing a C15 isoprenoid (namely 12-oxo-4,8-dimethyl-trideca-4,8-diene-1-al, ODTD) as a *single* major product, while RoxB and Lcp form a mixture of oligoisoprenoids of different length (C20, C25, C30, and larger) [274,275,276,277,278,279,280]. Hence, RoxA catalyzes processive oxidations starting from one end of the substrate with an *exo*-type action, and the pocket volume strictly governs the length of the product. RoxB and Lcp, instead, oxidize randomly NR at different locations with an *endo*-cleavage mode of action. Since Lcp and RoxB/A are not evolutionarily related, it means that polyisoprene-degrading pathways have evolved independently in different species.

RoxA from *Xanthonomas* sp. has been structurally characterized and shows an unusually low content of secondary structure elements (Figure 12a) [281]. Indeed, 67.3% of the protein structure is represented by loops, only 31.6% by helices, and the rest by a two-strended β-sheet. RoxA contains two c-type heme groups, one (heme1) in the N-terminal and one (heme2) in the C-terminal region, that are spaced by 21.4 Å and that feature different midpoint redox potentials (−65 and −130/−160 mV, respectively) [281,282]. The two heme cofactors are covalently linked to the protein via two cysteine residues each (Cys191 and Cys194; and Cys390 and Cys393, respectively) belonging to two distinct heme-binding motifs. Heme1 is the catalytic center and binds His195 as the proximal axial ligand and one dioxygen molecule as a stable distal axial ligand (as indicated by both structural and spectroscopic data). Heme2, instead, is less important for activity and His641 and His394 serve as proximal and distal axial ligands for its Fe, respectively [282].

Phe residue (Phe317) in the proximity of the coordinated O_2_ molecule is predicted to stabilize the binding of the latter to heme1. Mechanistic insights are scarce since no structural information on the RoxA–substrate interaction is available and eventual hydrophobic tunnel(s) for substrate access are not clearly visible in the experimentally solved structure. However, three loops are found near the distal face of heme1 that are rich in hydrophobic residues with rotationally flexible side chains that are supposed to assist NR binding within the protein matrix via substrate-induced conformational changes [281].

As for RoxB, no 3D structure is available, but it reasonably shares the same fold with RoxA and, as the latter, features two heme-binding motifs in the C- and N-terminal regions. Since RoxB has an *endo*-type activity, it is supposed to have a more extended binding groove with respect to RoxA, since it would facilitate random oxidation of the NR polymer [13].

Lcp architecture is definitely different from the one of RoxA/B and, as indicated by the crystal structure of an Lcp from *Streptomyces* sp. K30 (the only one available), the protein core is characterized by a canonical globin fold formed by eight helices labeled from A to H in analogy with hemoglobin (Figure 12b) [284]. Both the N- and C-terminus are additional domains of three and six helices, respectively, with unknown function. In contrast to RoxA/B, only one type-b heme group is found in Lcp, and it is predicted to have a different coordination environment according to the conformation assumed by the whole protein. Indeed, the Lcp 3D structure has been solved in both a “closed” and an “open” conformation: in the former, Fe is axially bound to His198 and Lys167, while in the latter, Lys167 is not coordinated and the accessibility to the heme is increased. The open state has been obtained in the presence of imidazole, which, in fact, replaces Lys167 as the axial ligand in the 3D structure. The equilibrium between the two conformations has been also confirmed by EPR spectroscopy, since both low-spin (6-coordinated) and high-spin (5-coordinated) Fe centers can be detected, and the high-spin species disappears upon imidazole addition [284].

Mechanistically, it was first proposed that Glu148 in Lcp may abstract one proton from an allylic position of NR triggering the oxidation process [284]. However, this possibility has been ruled out by a subsequent computational investigation performed by Zhang and Liu at the QM/MM level [285]. The authors first modeled the Lcp-NR interaction by docking a four-unit NR substrate into the Lcp cavity (in the open state), and by refining the most reasonable and productive pose via MD simulations. Then, different catalytic pathways have been envisioned and computationally dissected, and the main results can be summarized as follows:

- The Fe(II)-O_2_ system can be better described as an open shell singlet Fe(III)-O_2_^−^ species, and the distal O atom of O_2_^−^ is predicted to be more reactive than the proximal one, as indicated by its larger spin density;
- Glu148 *cannot act as a base* since the proton abstraction from the polyisoprene allylic position is energetically prohibitive. Geometry optimizations of the Lcp-substrate system considering three models for the mutants E148A, E148Q, and E148H revealed that the distance between the O_2_ ligand and the double bond of the substrate (undergoing oxidation) increases, suggesting that although Glu148 is not directly involved in the reaction, it can *indirectly* control substrate positioning within the pocket for productive catalysis.
- The energy profiles for assumed reaction pathways have been calculated, accounting for the different spin states that may originate along catalysis. O_2_^−^ can react with either one carbon or the other forming the isoprene C=C bond and, in each case, catalysis can proceed by forming a dioxetane or an epoxide intermediate. Therefore, four pathways can be envisioned overall. The most likely one (i) involves the C carbon that is closest to the O_2_^−^ ligand and (ii) entails the formation of a dioxetane intermediate, with an overall activation barrier of 15.5 kcal/mol (Figure 12d).

Remarkably, both NR and vulcanized rubber can be also oxidized by laccases and peroxidases (such as MnP) that are able to trigger the cleavage of both sulfide bridges and C=C double bonds of poly(cis-isoprene) backbone via radical chemistry according to a mechanism that has not been computationally unraveled yet [20,286,287,288].

### 6.2. Laccases and Heme Peroxidases for Plastic Degradation

Plastic pollution is an emergency experienced by all different environments throughout the planet, but it is particularly urgent in relation to the marine environment. Indeed, plastics, at variance with natural polymers, such as rubber, are materials recalcitrant to degradation and thus can have a very long half-life in the environment. For example, in the case of polyethylene terephthalate, the half-life in the ocean is estimated to be longer than 2500 years [289].

Interestingly, metalloenzymes with ligninolytic activity (i.e., laccases and peroxidases) proved to be effective in plastic biodegradation, such as PE and nylon-6,6. As for laccases, LMS was successfully employed to degrade both PE and nylon-6,6 in a pioneering contribution by Fujisawe et al. [19]: in the case of PE, its molecular weight decreased by 88.3% after 3 days of treatment with LMS and 1-hydroxybenzotriazole as a mediator. Recently, the idea that laccases are able to degrade PE is gaining ground [290,291]. In the case of the actinomycete *Rhodococcus ruber*, laccase incubated with PE [292] yielded a reduction of 20% of the polymer average molecular weight. More recently, the transfer of carbon atoms from ^13^C-labeled PE was observed [293] demonstrating that such a microorganism can use PE as a source of biomass and electrons.

Finally, laccase from *Bacillus subtilis* is able to increase the heavy oil biodegradation efficiency of bacterial consortia by approximately 15% [294]. An interesting aspect of the PE degradation by laccases is that the substrate binding is mostly due to hydrophobic interactions involving PE aliphatic chains, while hydrogen bonds would form with the alcohol or ketone groups of the pre-oxidized part of the substrate (if present). Moreover, during the monoelectronic oxidation process, aliphatic carbon radicals should be produced. This hypothesis was recently suggested in the context of an investigation on *Rhodococcus opacus* (strain R7), which produces laccase-like enzymes in the presence of PE [18,295]. Moreover, LSM with a laccase from *Bacillus subtilis* was employed on UV-treated low-density PE film sheets, evidencing a 40% loss of their molecular weight [18].

MnP and LiP have been reported to be involved in PE/nylon-6,6 degradation too [232,296], although their activity has been poorly characterized if compared to that of laccases. In some cases, PE and/or nylon-6,6 degradation by different bacterial cultures or fungi has been ascribed to the action of extracellular peroxidases (MnP or LiP) [17,296,297,298,299,300,301]. The activity of peroxidases toward plastics can be stimulated by H_2_O_2_, but MnP is supposed to be active towards PE even in the absence of H_2_O_2_ if a proper surfactant is employed [302].

It is still to be ascertained whether (and how) laccases/peroxidases can oxidize C-C bonds or only pre-oxidized portions of hydrocarbons. Computational tools could be suited to address this point, but the lack of structural information regarding the metalloenzyme–plastic complexes makes the building up of reliable in silico models challenging. A recent molecular docking investigation was performed in an attempt to propose possible PE biodegradation pathways by both laccases and heme-peroxidases [208]. The docking of a C12 linear alkane, used as a PE model, suggested a possible favorable binding at laccase T1 and MnP/LiP active sites (occurring with similar binding energy in all cases), although the simple substrate used is quite far from a folded and more realistic PE model [303]. In the case of laccase, the authors hypothesized that during the PE oxidation process, H_2_O_2_ is produced instead of H_2_O, a speculation that fits with the recent observations made by Perna et al. [304] that lignin oxidation induces enough H_2_O_2_ production to activate LPMO.

## 7. Conclusions

In this review we have outlined the molecular modeling literature of the last 20 years in the field of metalloenzymes that process, in various ways, biopolymers and some synthetic polymers, focusing on reactivity and enzyme–substrate interaction. In this context, computational modeling techniques provided valuable tools for understanding fine phenomena at the molecular level: details on catalytic mechanisms and electron transfer processes have been elucidated using QM or multiscale approaches, while the binding between metalloenzymes and their substrates have been simulated and rationalized mainly using classical techniques, such as molecular docking and molecular dynamics. These last have pros and cons: indeed, they are quite inexpensive from a computational point of view but have limitations in terms of accuracy and precision. On the other hand, higher-level calculations are decidedly computationally more demanding but ensure greater accuracy.

The interaction between LPMOs and crystalline substrates of different nature and morphology has been analyzed in detail thanks to several elegant MD contributions, showing that such an approach can be suited to drive the design of mutants with a specific (regio)selectivity. MD simulations were also crucial in confirming the location of heme peroxidases’ binding site, stressing the important role of second and outer coordination spheres in redox metalloenzymes [305]. Molecular docking simulations have undoubtedly given a significant boost to the knowledge of substrate binding mechanisms in laccases: several contributions have characterized in detail the topology and the driving force of the binding as a function of the origin of the enzyme and of its possible point mutations.

QM calculations, alone or in combined schemes, such as QM/MM or QM/MM/MD, elucidated subtle details on the catalytic mechanism of LPMOs, such as the nature of the key intermediate for HAA from the substrate and the presence of a mechanistic link between oxygenases and peroxygenases activity. These techniques, if necessary, adopted in combination with molecular docking simulations, shed light on the role of relevant reducing agents in the context of electron transfer processes and their interaction with the enzyme. Multiscale approaches were also fundamental for the definition of an active long-range electron transfer path essential for the oxidation of large molecules at the enzyme surface of LiPs and VPs and for clarifying the mechanism of action of heme oxygenases towards rubber.

As a perspective, we would like to point out that additional high-level theory investigations, in the framework of DFT or hybrid schemes, may be needed in the case of laccases since a number of unresolved issues regarding the mechanism of substrate oxidation deserve to be further addressed. Furthermore, another key point that surely requires computational rationalization is the interaction between most of these metalloenzymes (except LPMOs) and their substrates modeled in their “realistic” polymeric form. This will be particularly relevant both for lignin and plastics oxidation, for which many (or even all) mechanistic details are still lacking. Computational studies on this issue, which represents one of the open and most ambitious challenges within the fields of enzymatic waste biomass valorisation and bioremediation will surely benefit from the boost provided by future experimental investigations.

## Figures and Tables

**Table 1:** Summary of the literature about molecular docking, MD, QM, or hybrid studies on LPMO interactions with substrates or reducing agents.

LPMO	PDB	Docking	MD, QM, Hybrid	Ref.
*Neurospora crassa* AA9	4D7U	Xyloglucan, cellotetraose, cellopentaose, cellohexaose		[101]
*Neurospora crassa* AA9	4D7U	Cellohexaose		[59]
*Phanerochaete chrysosporium* AA9	4B5Q		MD with crystalline cellulose (1β)	[102]
*Heterobasidion irregulare* AA9	5NNS		MD with crystalline cellulose (1β)	[103]
*Serratia marcescens* AA10	2BEM		MD with crystalline chitin (α[100], α[110], β[100])	[104]
*Neurospora crassa* AA9	4EIR	CDH		[105]
*Four Neurospora crassa* AA9	4D7U, 4EIR, 4QI8, 4EIS	Five CYT structures *	MD, Umbrella sampling	[106]
*Lentinus similis* AA9	5ACF	CDH	MD, QM/MM/metadynamics	[107]
*Lentinus similis* AA9	5ACF	Ascorbate	QM/MM/metadynamics	[67]

* *Neurospora crassa* CDHIIA CYT (4Q1/), *Crassicarpon hotsonii* (*syn. Myriococcum thermophilum*) CYT (4Q13), *Phanerochaete chrysosporium* CYT (1D7B), *Neurospora crassa* CDHIIB CYT (homology model), *Crassicarpon thermophilum* (*syn. Corynascus thermophilus*) CDHIIB CYT (homology model).

**Table 2:** Residues involved in the laccase–substrate binding in the PDB crystallographic structures with a ligand. In bold are the His residues, which are bound to the T1 Cu site.

Laccase	PDB	Ligands	Residues	Ref.
*Bacillus subtilis*	4Q8B	Sinapic acid	**H419, H497** P226,R416,G417,G376,T337, T415, L386, 3H_2_O (see Figure 6a,b)	
*Bacillus subtilis*	1OF0	ABTS	**H419, H497** G417,T260, T415,L386	
*Trametes versicolor*	1KYA	2,5-xylidine	**H458** D206,F162, F265,L164,G392,P391 1H_2_O	[147]
*Bacillus subtilis*	3ZDW	ABTS	**H497** F226,F384,C229, C322, G323,A227	[148]
*Trametes trogii*	2HRG	p-methylbenzoate	**H455** A205,G391,V162,F331 1H_2_O	[149]
*Melanocarpus albomyces*	3FU9	2,6 dimethoxyphenol	**H508** F371,F427,L363,L429	[150]
*Pycnoporus sanguineus*	5NQ8	Phenol	**H477** F183,F353,H_2_O	[151]
*Zea mays* *(Plant Laccase)*	6KLI	Sinapic alcohol	**H519** E449,V348 N273 L446 G447 R518 D162 V276, W522, P362 G361	[152]
	6KLJ	Coniferyl alcohol	**H519** E449,Val348 N273 L446,L367,R518 D162 V276, W522, P362 G361,A360,H_2_O	[152]
*Bacillus subtilis*	4YWN	ABTS	Novel binding site far from T1	[153]

**Table 3:** Summary of the literature of molecular modeling studies on the interaction between lignin-related substrates or mediators with laccases.

Laccase	PDB	Docking	MD, QM, Hybrid	Ref.
*Trametes hirsuta Q02497*	Homology model	2,5-xylidine and syringaldehyde		[182]
*Rigidoporus lignosus*	1V10	2,5-xylidine		[183]
*Trametes versicolor*	1KYA wt and F162A, F332A	2,5-xylidine, 3,5-Di-*t*-Bu-phenol, and 2,6-di-*t*-Bu-phenol		[184]
*Trametes versicolor*	1GYC	Di-lignol model	MD	[185]
*Trametes versicolor*	Four isoforms	ABTS	MD	[186]
*Trametes versicolor*	1GYC	Di- and tri-lignols		[187]
*Pycnoporus cinnabarinus*	2XYB	ABTS and 2,6-dimethoxyphenol	QM/MM	[188]
*Trametes versicolor*	1GYC	Monolignols, 2,6-dimethoxyphenol, ferulic acid, guaiacol, sinapic acid, and vanillyl alcohol		[189,190]
*Populus trichocarpa* *(plant laccase)* *Trametes versicolor* *(fungal laccase)*	1AOZ 1KYA	Mono-, di-, tri- and tetramer lignin models		[168]
*Homologus chimeric 3A4 laccase from basidiomycetes PM1*	5ANH	Sinapic acid,methyl syringate, dehydrodisinapic acid, and dilactone		[191]
*Homologus from* *Trametes versicolor*	1KYA	Aflatoxin B1 and M1		[192]
*Myceliophthora thermophila* *Pycnoporus cinnabarinus*	6F5K 2XYB	Syringaldazine and four phenols	QM/MM	[193]
*Trametes versicolor*	1KYA	2,6 dimetoxy phenol	MD	[194]
*Trametes versicolor* *Cerrena unicolor*	1GYC and homology model	Sinapic acid, syringaldazine, 2,6-dichlorophenol, hyrocaffeic acid, catechol, 2.6-dimethoxyphenol, ABTS, n-(1-Naphtyl) ethylendiamine, 3-amino-4-hydroxy benzenesulfonic acid, and 2-methoxyhydroquinone	MD	[195]
*Trametes versicolor*	1GYC	54 substrates from [137,196]	DFT	[132]
*Trametes versicolor*	1GYC	Nonylphenol and octylophenol isomers		[197]
*Six fungal laccase sequences*	Homology model	Mono-, di-, tri-, and tetramer lignin models		[198]
*Trametes versicolor*	1GYC	Phenol, surfactant triton X-100, and rhamnolipid	MD	[199]
*Coriolopsis gallica*	4A2E		MD QM/MM	[200]
*Cryptococcus neoformans*		Prostaglandin and phospholipids	MD	[201]
*Trametes versicolor*	1GYC	Bisphenol		[202]
*Trametes versicolor*	1GYC	Coniferyl alcohol		[175]
*Trametes versicolor*	1GYC	15 phenolic compounds	QM	[132]
*Ganoderma weberianum*	Homology model	2,4-dichlorophenol, benzidine, sulϐisoxazole, tetracycline, trimethoprim, ABTS, and 2,6-dimethoxyphenol		[203]
*Saccharomyces* *cerevisiae*	6H5Y	N,N-dimethyl-p-phenylenediamine, and 1-hydroxybenzotriazole		[169]
*Stropharia* sp. *ITCC-8422*	Homology model	ABTS, 2,6-dimethoxyphenol guaiacol, and syringaldazine		[204]
*Amylostereum areolatum*	Homology model	Mono-, di-, tri-, and tetramer lignin models		[205,206]
*Trametes* sp. *C30*	Homology model	Four aflatoxins		[207]
*Trametes versicolor*	1KYA	Dodecane		[208]
*Antrodiella faginea*	5EHF	Lignosulfonic acid		[209]
High, medium, and low redox potential laccase	2HZH (805 mV) 1KYA (780 mV) 1GYC (780 mV) 3FU7 (460 mV)	Lignin tetramer		[190]
*Five bacterial laccases*	Homology model	Guaiacol, ABTS, and DMP	MD	[189,210]
*Cryptococcus neoformans*	Homology model	Ellagic acid	MD	[211]
*Comamonas testosteroni*	Homology model	Mono-, di-, tri-, and tetramer lignin models		[212]
*Trametes versicolor*	1KYA	Glyphosate, isoproturn, dilignol model, and parathion	MD	[213]
*Trametes versicolor*	1KYA	Two aflatoxins	MD	[214]

## Glossary

**MM***:* Molecular mechanics is a technique employed in molecular modeling to investigate the properties of molecules, such as their stability, geometry, and energy, using classical mechanics principles. MM is a method based on classical physics for calculating the potential energy surface (PES) of a molecular system in terms of “Force Field” (FF). In a nutshell, FF evaluates the internal energy U of polyatomic systems (in which atoms are considered as point-like masses) as a sum of various contributions arising from bonded elastic terms, such as covalent bonds, bond angles torsions, and non-bonded terms, such as long range electrostatic and van der Waals interactions. All contributions to the total energy U are based upon parameters, which can both be empirically determined (e.g., equilibrium distances in given covalent bonds are taken from XRD and/or NMR) and be computationally obtained (e.g., atomic partial charges necessary for electrostatic long-range interactions can be obtained by DFT methods, vide infra). MM is essentially aimed at pinpointing the *optimal* conformation of a molecule, corresponding to the global minimum on the PES (or other relative minima that may correspond to elusive intermediates along catalytic cycles, for example). This method is especially helpful for studying molecular systems where quantum mechanical calculations are not viable. **MD***:* Molecular dynamics is a computational method that simulates the dynamic behavior of molecular systems by applying classical mechanics and statistical thermodynamics principles [306]. In these simulations, the atomic trajectories, i.e., movements of individual particles over time are determined by solving Newton’s equations of motion. The forces governing the reciprocal interaction between particles are normally evaluated by means of FF potentials (see MM). MD is widely used in molecular modeling to examine a range of phenomena, including protein dynamics, protein–ligand interactions, and material properties. **Umbrella sampling***:* Umbrella sampling MD, developed by Torrie and Valleau in 1977 [307], is a method used to calculate free energy changes along a reaction coordinate. In this technique, a system is driven from one thermodynamic state to another using bias potentials along the reaction coordinate, and MD simulations are carried out at multiple windows to cover the intermediate steps. The bias potentials can take on various functional forms with harmonic potentials being the most commonly used due to their simplicity. By analyzing the sampled distribution of the system along the reaction coordinate, the change in free energy in each window can be determined. Finally, the windows are combined using techniques, such as umbrella integration or the weighted histogram analysis method (WHAM) [308]. **Metadynamics***:* Metadynamics (MetaD) is an advanced sampling method in molecular simulations that enables the reconstruction of the free energy landscape as a function of collective variables (CVs) [309]. By applying a history-dependent bias potential that is a function of selected CVs, MetaD enhances sampling and discourages revisiting previously sampled configurations. The bias potential consists of Gaussian terms for each CV, which push the system away from local free energy minima and facilitate the exploration of rare events. Overall, MetaD is a powerful technique for efficient sampling of complex systems in molecular simulations. **Molecular docking***:* Molecular docking is a computational technique used to predict how two generic molecules will interact with each other to form a stable complex [310]. It may use (but other strategies may be adopted as well) principles of MM (*vide supra*) to calculate the energy of the interactions between a given ligand (that can be a small molecule, typically synthetic organic compounds) and the receptor (typically a protein or other macromolecules). The interaction energy between the two molecules is referred to as “Scoring Function” and is used to establish a ranking of poses, namely mutual conformational arrangements (or *binding modes*) of the two interacting molecules. Various (and different) algorithms are employed to sample the phase space (the whole set) of the possible poses, such as Metropolis–Hastings or Monte Carlo methods [311,312]. Molecular docking can be used also to predict the formation of protein-protein complexes. **QM (DFT + CASPT2)***:* Density functional theory (DFT) is a computational method used in quantum chemistry to study the electronic structure of molecules and materials [313]. It is based on the principle that the total ground-state energy of a system can be expressed as a functional of the electron density, which is a mathematical function that describes the probability of finding an electron in a particular region of space [314]. This approach diverts from wave function-based methods, all conceiving the ground-state energy as the expectation value arising from a variational procedure (a minimization scheme) applied to parameters, such as wave function or atomic-orbital based combinations. Complete Active Space Second Order Perturbation Theory (CASPT2) is the application of perturbation theory to the post Hartree–Fock multi-references CAS (Complete Active Space) wave function, as developed by Roos and coworkers [315], that allows to accurately describes the electronic states of molecules. **QM/MM***:* Quantum Mechanics/Molecular Mechanics is a hybrid computational approach that combines quantum mechanics and classical molecular mechanics to study the properties and behavior of chemical systems [314,315,316]. In QM/MM calculations, the region of interest that, for instance, is involved in a chemical process (such as an enzyme active site) is treated at the QM level. Conversely, the surrounding environment, which may consist of a large number of atoms and solvent molecules, is treated at the MM level. **QM/MM/MD**: Quantum Mechanics/Molecular Mechanics/Molecular Dynamics is a multiscale computational approach that combines QM/MM and MD to study the behavior of molecular systems [316]. QM/MM/MD is a promising tool to explore chemical reactions that simultaneously allows an extensive conformational sampling and an explicit description of electronic structure variation occurring in a chemical transformation.
